# Intravitreal AAV vector delivery induces integrin-dependent ocular inflammation, complement activation, and antiviral and DNA damage responses

**DOI:** 10.3389/fimmu.2026.1799327

**Published:** 2026-04-22

**Authors:** Helena Costa-Verdera, Deepa Jamwal, Emily Fabyanic, Ariel Hippen, Madeleine Salvatore, Aiping Du, Anna Majowicz, David Markusic, Jon E. Chatterton, Virginia Haurigot, Klaudia Kuranda

**Affiliations:** 1 Immunology, Spark Therapeutics, Philadelphia, PA, United States; 2 Ocular Research, Spark Therapeutics, Philadelphia, PA, United States; 3 Genomics and Data Science, Spark Therapeutics, Philadelphia, PA, United States; 4 Non-clinical Research Operations, Spark Therapeutics, Philadelphia, PA, United States

**Keywords:** AAV gene therapy, antiviral immunity, DNA damage response, integrins, intravitreal AAV injection, ocular gene transfer, uveitis

## Abstract

**Background:**

Intravitreal delivery of adeno-associated virus (AAV) vectors offers a promising, minimally invasive strategy for retinal gene therapy, but remains limited by dose-dependent intraocular inflammation. Corticosteroid therapy, the current standard for managing gene therapy-associated uveitis (GTAU), can be ineffective or contraindicated, highlighting the need for alternative immunomodulatory approaches and deeper understanding of AAV-induced inflammation.

**Methods:**

Using porcine and mice models, we characterized immune responses triggered by AAV2.7m8 vector.

**Results:**

Consistent with previous data for this serotype, AAV-mediated transgene expression localized predominantly to retinal ganglion cells, although photoreceptor, bipolar, amacrine, horizontal, and glia cells were also transduced. Despite prophylactic methylprednisolone, AAV-treated animals developed GTAU, accompanied by increased MCP-1, IP-10, MIP-1α and IL-6 levels in ocular humors, along with microglial activation and peripheral leukocyte infiltration. Transcriptomic analysis revealed upregulation of antiviral interferon responses across all retinal cell populations, together with complement and DNA damage pathways. Accordingly, functional assays confirmed complement C3a accumulation in ocular humors and presence of γH2AX^+^ DNA damage foci in transduced retinas. Finally, a mechanistic study of intravitreal AAV delivery in mice using integrin-blocking antibodies revealed a role for peripheral leukocytes in mediating ocular inflammation in this species.

**Conclusion:**

Our work identified new markers of ocular inflammation and potential targets to modulate GTAU.

## Introduction

Adeno-associated virus (AAV)-based gene therapy has the potential to achieve higher and more durable transgene expression than non-viral methods, with low immunogenicity and a favorable safety profile (1). Moreover, unlike integrating vectors, AAV genomes remain largely episomal, minimizing the risk of insertional mutagenesis (2). In preclinical models, intra-ocular AAV delivery has shown to induce long-term transgene expression, ranging from months to years (3). Sustained clinical expression after a single administration has been documented in patients with inherited retinal disease (4). For this reason, AAV vectors are particularly well suited for monogenic disorders affecting post-mitotic tissues, especially in inherited retinal dystrophies such as Leber congenital amaurosis and retinitis pigmentosa, where long-term gene expression in non-dividing cells is required (5).

The healthy eye is an immune-privileged site in which inflammation is restrained by the blood–retinal barrier, low MHC expression, and a local immunosuppressive milieu, while resident microglia and perivascular macrophages provide regulated immune surveillance. In uveitis, barrier breakdown enables infiltration of activated T cells, B cells, and monocytes, resident myeloid cells shift to a pro-inflammatory state, and cytokines drive persistent intraocular inflammation and tissue damage (6). AAV vector administration to the subretinal space has been linked to a phenomenon similar to anterior chamber-associated immune deviation (ACAID) (7–9), which promotes immunological tolerance to antigens targeted to the retina, and does not trigger anti-AAV humoral responses (10, 11). On the other hand, intravitreal (IVT) injection is a less invasive but more immunogenic route, associated with dose-dependent intra-ocular inflammation (IOI) that compromises the long-term efficacy of gene transfer (11–13).

AAV vector delivery via intravitreal (IVT) injection is a minimally invasive procedure that offers the potential for broad inner retinal transduction, providing significant transgene expression in the ganglion cell layer (GCL) (10, 14–17), and in particular in retinal ganglion cells (RGCs) (17–20). Accordingly, IVT-administered AAV gene therapies are under development for conditions where: *(i)* RGCs are compromised, such as glaucoma (21, 22) or Leber hereditary optic neuropathy (LHON) (18, 23); (*ii)* secretion of the therapeutic protein into the vitreous humor is required, as in wet age-related macular degeneration (wAMD) (3) or diabetic retinopathy (24); or (*iii)* an alternative to subretinal AAV administration is necessary for individuals with progressive retinal degeneration, including retinitis pigmentosa or Leber congenital amaurosis (23). AAV serotypes used for IVT gene transfer include AAV2 (17–20) and its engineered version, AAV2.7m8, which offers improved retinal penetration (25, 26). Thanks to its efficient transduction, AAV2.7m8 is currently being evaluated in the clinic for the treatment of neovascular AMD (nAMD) (27).

Athough IVT AAV administration is a minimally invasive, office-based procedure in contrast to subretinal delivery, it is linked to dose-dependent intraocular inflammation (IOI) and gene therapy-associated uveitis (GTAU) (28, 29). GTAU poses concerns for the long-term efficacy of gene transfer and is linked to potential loss of retinal cells and subsequent deterioration of visual function due to sustained inflammation. The standard of care for managing GTAU includes local and/or systemic corticosteroid therapy. However, corticosteroids are contraindicated in certain patients due to the risk of elevated intraocular pressure. Additionally, persistent inflammation has been observed in patients receiving high doses of both AAV2 or AAV2.7m8, despite steroid treatment (27, 30). IVT dosing of AAV2.7m8 has also been linked to severe, dose-limiting toxicities and vision loss at the high dose of 6×10^11^ vector genomes (vg)/eye, leading to discontinuation of Adverum’s INFINITY clinical trial for retinal macular edema (31). To overcome the challenges of AAV-induced toxicity and immunogenicity, which can potentially be exacerbated by underlying disease-associated inflammation, it is necessary to understand the mechanisms underlying GTAU and to identify novel alternative immunomodulation targets.

Preclinical investigations utilizing mice have limited utility in predicting severe adverse events, as outcomes from AAV2 IVT injections without immunomodulation predominantly reveal mild to moderate inflammation accompanied by ocular infiltration of CD45^+^ peripheral immune cells, including T cells (30, 32–34). On the other hand, studies in non-human primates (NHPs) have shown that IVT AAV2 administration at the dose of 5×10^11^ vg/eye can induce uveitis in the presence of prophylactic systemic methylprednisolone (30). Given the greater accessibility of pigs compared to NHPs, we aimed at assessing immune responses to IVT AAV-mediated gene transfer in this large preclinical model, employing the clinically relevant serotype AAV2.7m8. The pig eye is extensively used for cornea, lens and retinal research. Additionally, due to the similarity in size and anatomy to the human eye, pigs are useful models for development of surgical procedures and testing of subretinal and intravitreal dosing of therapeutic drugs. Furthermore, the pig immune system is estimated to be 80% similar to human (35). Thus, the pig offers a wide range of established methods for assessing IOI in response to local administration of gene therapy vectors.

Here we show that IVT AAV2.7m8 injection in pigs delivered the vector genome to all retinal layers, with the highest transgene expression in RGCs. Despite prophylactic immunosuppression, we observed secretion of pro-inflammatory cytokines to ocular humors, complement activation, broad formation of DNA-damage foci, accumulation of reactive microglia in transduced retinas, as well as activation of transcriptional antiviral interferon (IFN) responses. Additionally, IVT AAV injections were followed by peripheral CD45^+^ cell infiltration into ocular tissues. To investigate whether these infiltrating cells play an active part in IOI, we performed a study in mice to test whether blocking their entry would affect inflammation. For this purpose, we used dual blockade of integrins with anti-leukocyte function-associated antigen-1 (LFA-1) and anti-very late antigen-4 (VLA-4) neutralizing antibodies. Integrin blockade not only prevented significant ocular infiltration by peripheral leukocytes but also reduced inflammation and increased transgene expression levels. Thus, our work identified specific signaling pathways and cell types as potential targets for novel immunomodulation strategies to improve safety and efficacy of IVT AAV-based gene transfer.

## Materials and methods

### Viral vectors

AAV2.7m8/CAG-GFP and AAV2.7m8/CAG-FIX were produced by standard transient triple transfection (36) in HEK293 adherent culture. Full particles were purified by double CsCl density gradient ultracentrifugation, which is reported to render vector preparations with >90% full capsid (37). Purified AAVs were formulated under sterile conditions in buffer containing 180mM Sodium Chloride, 10mM Sodium Phosphate, 0.001% Pluronic F68, pH 7.3. Vector titer was determined by qPCR as vg per mL using primers directed against the transgene poly-adenylation signal region. The AAV titer was calculated by averaging results from 2 qPCR rounds with three different dilutions tested for each sample in each round. Vector titers were 3.68×10^12^ vg/mL for the AAV2.7m8/CAG-GFP vector, and 8.81×10^12^ vg/mL for the AAV2.7m8/CAG-hFIX vector. For both vectors, endotoxin levels were below 0.100 EU/mL. The purity of vector preparations was evaluated by SDS-PAGE gel identifying distinct VP1, VP2 and VP3 bands.

### Intravitreal AAV injections in pigs

The protocol was approved by the Institutional Animal Care and Use Committee (IACUC # PR-206) of Powered Research, Durham, NC. Female Yucatan minipigs of 3–5 months old were purchased from Premier Biosource and pre-screened for anti-AAV2.7m8 neutralizing antibodies. All animals were housed on a 12:12 h light–dark cycle, and the temperature in housing rooms was 20-25 °C. Animals were fed Laboratory Porcine Grower Diet (PMI 5084, Lab Diet) 1 scoop twice daily, and water was available ad libitum.

Animals received a 50 µL/eye 200 USP/mL intravitreal (IVT) injection of Vitrase in both eyes two weeks prior to AAV injection, to facilitate vector diffusion through the vitreous. All animals were pre-treated with intramuscular methylprednisolone (5 mg/kg) two days prior to AAV dosing, and then weekly for the duration of the study. On day 0, animals received 100 µL/eye bilateral IVT injection of 5x10^11^ vg/eye AAV2.7m8/CAG-GFP (n = 7) or vehicle buffer (n = 5). Aqueous humors (AH) were collected at baseline, days 8, 15 and terminal timepoint. Vitreous humors (VH) were collected at terminal timepoint from eyes not used for immunofluorescence.

All animals underwent *in situ* perfusion at each terminal timepoint prior to collection of ocular tissue samples. N = 1 AAV-injected and n = 1 vehicle control animals were sacrificed on day 15, and all eyes were used for snRNAseq. 3 AAV-injected and 2 vehicle controls were sacrificed on day 28. Due to the detection inflammation, 3 AAV-injected and 2 vehicle controls were sacrificed prematurely on day 24, to avoid the use of corticosteroids that would interfere with the results. Eyes from AAV-injected and vehicle groups were distributed for assays on AH and VH, snRNAseq and immunofluorescence imaging uses. Finally, another cohort received bilateral IVT injections of 5x10^11^ vg/eye of AAV2.7m8/CAG-FIX (n = 3) or vehicle (n = 1) and sacrificed on day 28. One AAV-injected animal received topical corticosteroids on day 8 post-injection due to early detection of inflammation, in order to complete the study. Eyes were used for MACSima imaging and flow cytometry.

For anesthesia during intravitreal AAV dosing in pigs, animals received first a topical mydriatic (1.0% tropicamide HCl). Then buprenorphine (0.01-0.03 mg/kg) and atropine (0.05 mg/kg) were administered intramuscularly. Animals were anesthetized with intramuscular ketamine (10–15 mg/kg) and dexmedetomidine (0.05 – 0.3 mg/kg), with the addition of acepromazine (0.1-0.5 mg/kg) to ease the recovery of the animals. Prior to euthanasia, anesthesia was performed with intramuscular ketamine (10 mg/kg) and dexmedetomidine (0.05 mg/kg) with addition of buprenorphine (0.05 mg/kg). Animals were euthanized with an overdose of 150 mg/kg sodium pentobarbital administered IV, followed by auscultation to ensure death.

### Intravitreal AAV injections in mice

The protocol was approved by the Institutional Animal Care and Use Committee (IACUC # PR-117 A15) of Powered Research, Durham, NC. Female C57Bl/6J mice (8–12 weeks) were purchased from Jackson Laboratories. All animals were housed on a 12:12 h light–dark cycle, and the temperature in housing rooms was 20-25 °C. Animals were fed Lab Diet 5K52. Food and water were available ad libitum. Mice were enrolled into 3 groups. On day 0, group 1 (n = 20) received bilateral IVT injections of vehicle solution in 1 µL injection volume. Groups 2 (n = 19) and 3 (n = 10) received a 1 µL bilateral IVT injection of AAV2.7m8/CAG-GFP at 1x10^9^ vg/eye. Additionally, group 3 animals received intraperitoneal (I.P.) injections of anti-VLA-4/LFA-1 antibodies (both from *In vivo*Mab) at 0.5 mg/mL in 200 µL injection volume, on day 0 and every 3 days until termination on day 28. Color and cobalt blue fundus imaging was performed at baseline and weekly to monitor GFP fluorescence. At the indicated timepoints animals were sacrificed, and left ventricular perfusion was performed using ice-cold heparinized PBS. VH was removed from all eyes and frozen, and fresh ocular globes were collected into cold RPMI/HEPES for flow cytometry processing. For anesthesia during intravitreal AAV dosing in mice, animals received buprenorphine (0.01-0.05 mg/kg) subcutaneously for pain management. At least fifteen minutes prior to anesthetic administration, a cocktail of 1% tropicamide HCl and 2.5% phenylephrine HCl (Tropi-Phen) was applied topically to dilate and proptose the eyes. Animals were sedated immediately prior to the procedure with a ketamine/xylazine cocktail (80-90/10–20 mg/kg) administered intraperitoneally. For euthanasia, animals were tranquilized with a ketamine/xylazine cocktail (80-90/10–20 mg/kg) administered intraperitoneally. Animals were euthanized via exsanguination and death was confirmed by thoracotomy.

### Live ocular examinations and imaging

At baseline and on weeks 1, 2 and 4, AAV/GFP-injected animals underwent color and cobalt blue fundus imaging for GFP visualization. Fundus imaging was performed with the optic nerve head oriented centrally. The Phoenix Micron III microscope was used to visualize GFP fluorescence over time, and quantification of GFP MFI was determined with ImageJ software (version 1.54p). In pig studies, fundus imaging was performed with RetCam^®^ 3 imaging system (Natus). Ocular examinations were performed at baseline to determine enrollment, and at the indicated timepoints to evaluate toxicologic or inflammatory changes, using a slit lamp biomicroscope and indirect ophthalmoscope. The semiquantitative preclinical ocular toxicology scoring (SPOTS) ocular grading system (38) was used for uveitis scoring in pigs. Refer to **Supplementary Tables 1**–**4** for detailed scores. Optical coherence tomography (OCT) with blue autofluorescence (BAF) with enhanced depth imaging was performed using a Spectralis HRA OCT II (Heidelberg Engineering) to evaluate anterior and posterior segment integrity.

### Anti-AAV IgG ELISA

The concentration of anti-AAV2.7m8 IgG in AH and VH was determined by enzyme-linked immunosorbent assay (ELISA). Purified empty AAV2.7m8 capsid was used to coat 96-well MaxiSorp Nunc-immunoplates (VWR, Radnor, PA) at 1x10^9^ vg/well in 50µl per well (2x10^10^ vg/ml) diluted in ELISA coating buffer (Biolegend, #421701). For standard curve, purified IgG (Southern Biotech, Swine IgG-UNLB # 0137-01) was serially diluted in coating buffer and plated in 50µl per well. Plates were incubated overnight at 4°C. Next, plates were washed 3 times with 200µl PBS-tween 0.05%, and plates were blocked for 2h at room temperature with 200µl PBS-Tween 0.05% - BSA 2%. After blocking, AH and VH were diluted 1:100 or 1:500, and 50µl of each dilution were added per well to wells coated with AAV. Diluent buffer was added to wells coated with IgG, in duplicates. Plates were incubated for 1h at 37°C. Plates were again washed as previously and 50µl of Goat Anti-Porcine IgG(H+L)-HRP (Southern Biotech, #6050-05) were added to all wells. After 1h incubation at 37°C, plates were washed, and revelation was performed by short incubation with 100ul TMB substrate (Abcam, #ab210902) followed by 100µl of stop solution (Abcam, ab171529). Absorbance was measured at 450nm with Promega™ GloMax Plate Reader.

### Analysis of complement C3a and cytokines in ocular humors

Cleaved C3a levels in AH and VH ocular humors were assessed with the Pig Complement Component 3a (C3a) ELISA Kit (BIOMATIK, #EKU03389) following the kit instructions. AH and VH samples were diluted 1:100 in diluent for C3a detection prior to addition to pre-coated wells. Absorbance was measured at 450nm with Promega™ GloMax Plate Reader. The concentration of cytokines MCP-1 (CCL2), IP-10 (CXCL10), interleukin 6 (IL-6) and MIP-1α in ocular humors was assessed by MSD custom U-plex (Mesoscale Discovery, MSD, #K15235N-2). Biotinylated antibodies against all four porcine analytes were coated into the same well according to the manufacturer’s instructions. Standards, capture and detection antibodies were purchased from King Fisher Biotech (**Supplementary Table 6**). Primary antibodies used for detection were labelled with MSD gold sulfo-tag NHS-Ester Conjugation kit (Mesoscale discovery, MSD, #R31AA) prior to use. Samples were diluted 1:2 in 25µl per well in 96-well plates and incubated overnight at 4°C prior to detection. Plates were read with MESO SECTOR S 600 instrument (MSD). Results were analyzed with MSD discovery workbench (MSD).

### Immunofluorescence microscopy

After fixation with 4% PFA, frozen whole eyes were bisected as temple and nasal sides, vitreous was removed and globes were embedded in OCT freezing medium. Fixed ocular globes were sectioned at 7µM, and slides were loaded onto the MACSima Imaging System four-well cassette (Miltenyi Biotech). Slides were subsequently stained with DAPI for 10 min and washed thrice with MACSima running buffer (Miltenyi Biotech). Tissues were then stained and imaged in a cyclical manner through rounds of staining with fluorescently labelled primary antibodies and subsequent bleaching in the MACSima instrument. MACSima images were analyzed with MACS^®^ iQ View Analysis Software (Miltenyi Biotech). Refer to **Supplementary Table 6** for details on primary antibodies, all used at 1:50 dilution.

RNAscope was performed on ocular cryosections prepared as before. RNAscope^®^ *in situ* hybridization was performed using the RNAscope^®^ LS Multiplex Fluorescent Reagent Kit (Advanced Cell Diagnostics, ADC, #322800) on the Leica BOND RX automated stainer, according to the ACD’s protocol. Tissue sections were post-fixed in 10% neutral buffered formalin (NBF) for 30 minutes prior to processing. Target-specific probes for CAG-promoter-O4-sense-C1 (ACD, #1280358-C1) were used to detect vector DNA. Following hybridization, signal amplification was performed using the standard AMP1–AMP3 steps included in the kit. Fluorescent detection was carried out using Opal 570 for CAG (Akoya Biosciences), applied per ACD’s recommended protocol for multiplex detection. DAPI was used as a nuclear counterstain. Images were acquired with Zeiss Axioscan Z7 (Zen v8.8) and viewed with HALO Link software v4.05.

### Flow cytometry

After removing optic nerve, cornea and lens, freshly isolated whole ocular globes were dissociated using the Miltenyi Biotec Neural Tissue Dissociation Kit – Postnatal Neurons (Miltenyi, #130-094-802). First, globes were transferred to a petri dish with RPMI with 2% Fetal bovine serum (FBS) placed on ice. Optic nerve, cornea, and lens were removed, followed by one PBS wash. PBS was discarded, and enzyme mix 1 was added. Dissected ocular globes were then minced into small pieces in the solution, and all the material was transferred to a new Falcon tube to proceed to incubation steps and addition of enzyme mixes as indicated in the protocol. Finally, tissue homogenates were applied to a MACS^®^ SmartStrainer (70 μm) placed on a 50 mL tube, and 10mL of RPMI media supplemented with 2% FBS were added immediately. A syringe plunger was used to further dissociate the tissue. Additional cold RPMI + 2% FBS was used to wash the filter, and single cells were pelleted by centrifuging tubes at 400×g for 15 minutes. The supernatant was removed carefully, and cells were gently resuspended in cold RPMI with 2% FBS using a 1 mL pipette. Cells were then counted and immediately processed for staining and flow cytometry.

Cells were transferred to a V-bottom 96-well plate with lid, pelleted and resuspended in PBS 0.5% BSA containing porcine Fc block (Thermo Fisher, #14-9161-73). After 15min incubation on ice, surface staining was performed with primary antibodies listed in **Supplementary Table 6**. After 30 min incubation on ice, cells were washed and incubated for 15min with Zombie Aqua™ Fixable Viability Kit (Biolegend, #423101). Finally, cells were washed and resuspended in sterile PBS. Cells were acquired on a Cytoflex flow cytometer (Beckman Coulter). Data analysis was performed using Flowjo software v10.9 (Tree star, Ashland, OR, United States).

### Single-nucleus RNAseq

#### Nuclei isolation and sequencing

Two eyes per condition, vehicle Week 2 and week 4, and AAV2.7m8/CAG-GFP week 2 and week 4, were processed individually for nuclei extraction and sequencing. After recovering terminal AH and VH samples, whole globes were flash frozen via liquid nitrogen submersion. Following freezing, retinas were dissected out and frozen. Nuclei were isolated from frozen tissue using the 10x Genomics Nuclei Isolation Kit with RNase Inhibitor (10x Genomics) with minor modifications. First, flash-frozen tissue was transferred to ice-cold lysis buffer (from the Nuclei Isolation Kit) in a 1mL glass tissue homogenizer (Wheaton). After douncing, the tissue lysate was resuspended and left to incubate for 10min on ice. Additional steps followed the manufacturer’s instructions. During the first wash (after debris removal), the nuclear suspension was incubated with rotation for 20min at 4°C with DAPI staining solution (Abcam) at a final concentration of 5µM. Following a spin and final resuspension, nuclei were purified by fluorescence-activated nuclear sorting (Sony Cell Sorter) based on DAPI-fluorescence and collected in Wash and Resuspension Buffer (PBS, 0.5% BSA, and 0.2U/µl RNase Inhibitor - prepared according to kit instructions). Sorted nuclei event count and final volume was used to inform sample loading volume, and the nuclei were immediately loaded onto 10x Chromium GEM-X Single Cell 3’ Chip Kit v4, at ~20k nuclei per sample (this loading concentration was optimized based on sorter nuclear count and 10x nuclear recovery). Four lanes per sample were loaded to maximize cell recovery. Libraries were generated following the manufacturer’s protocol and were sequenced using the Illumina NextSeq 2000.

#### Data processing and graph-based clustering

The snRNAseq data was aligned to a custom reference containing both the pig genome (Sscrofa11.1, release 113) and the EGFP transgene. Gene expression counts were quantified using Cellranger version 8.0.0, from the Chromium Single Cell Software Suite by 10x Genomics. The resulting expression matrix was imported into R and analyzed with Seurat version 5.1.0. Cell filtering was performed using miQC (via the SeuratWrappers package version 0.3.1) to remove droplets with high mitochondrial contamination, suggestive of incomplete nuclear fractionation. Single-nucleus datasets from different samples were integrated using SCTransform version 0.4.2 to remove batch effects. The data was visualized using Uniform Manifold Approximation and Projection (UMAP) on the first 30 principal components. Cell clustering was performed using the Louvain algorithm with a resolution r=0.03 via the FindClusters function in Seurat. Differential expression analysis using the FindAllMarkers function in Seurat identified marker genes for each cluster. Each cell cluster was then manually annotated and assigned to a cell type based on known ocular marker genes.

Cells were then stratified based on cell type and time post-injection the sample was taken. Differential expression analysis was performed for each cell type and timepoint, comparing gene expression in cells of the given type from pigs that had been injected with transgene-containing AAV2.7m8/CAG-GFP vs. vehicle. To identify patterns in the differentially expressed genes, Gene Set Enrichment Analysis (GSEA) was performed with Hallmark genes from the Molecular Signatures Database (MSigDB) as the gene sets. This was done using the R package fgsea version 1.28.0, which uses a permutation-based approach and compares the enrichment scores to a null distribution of shuffled genes to test for significance.

### Statistical analysis

Data are reported as mean or mean ± SD as reported in the figure legends. GraphPad Prism 10 (GraphPad Software) was used for statistical analyses. P < 0.05 was considered significant. For multiple comparisons, statistical significance was determined by one-way ANOVA with Tukey’s *post-hoc* correction. For two group comparisons, statistical significance was determined by Mann-Whitney t-test or Welch’s t-test as indicated in the figure legend. For all figures, *p <0.05, **p <0.01, ***p <0.001, ***p <0.0001.

## Results

### Preliminary assessment of IVT gene transfer in pigs shows early inflammation post-AAV injection

**Figure 1:**
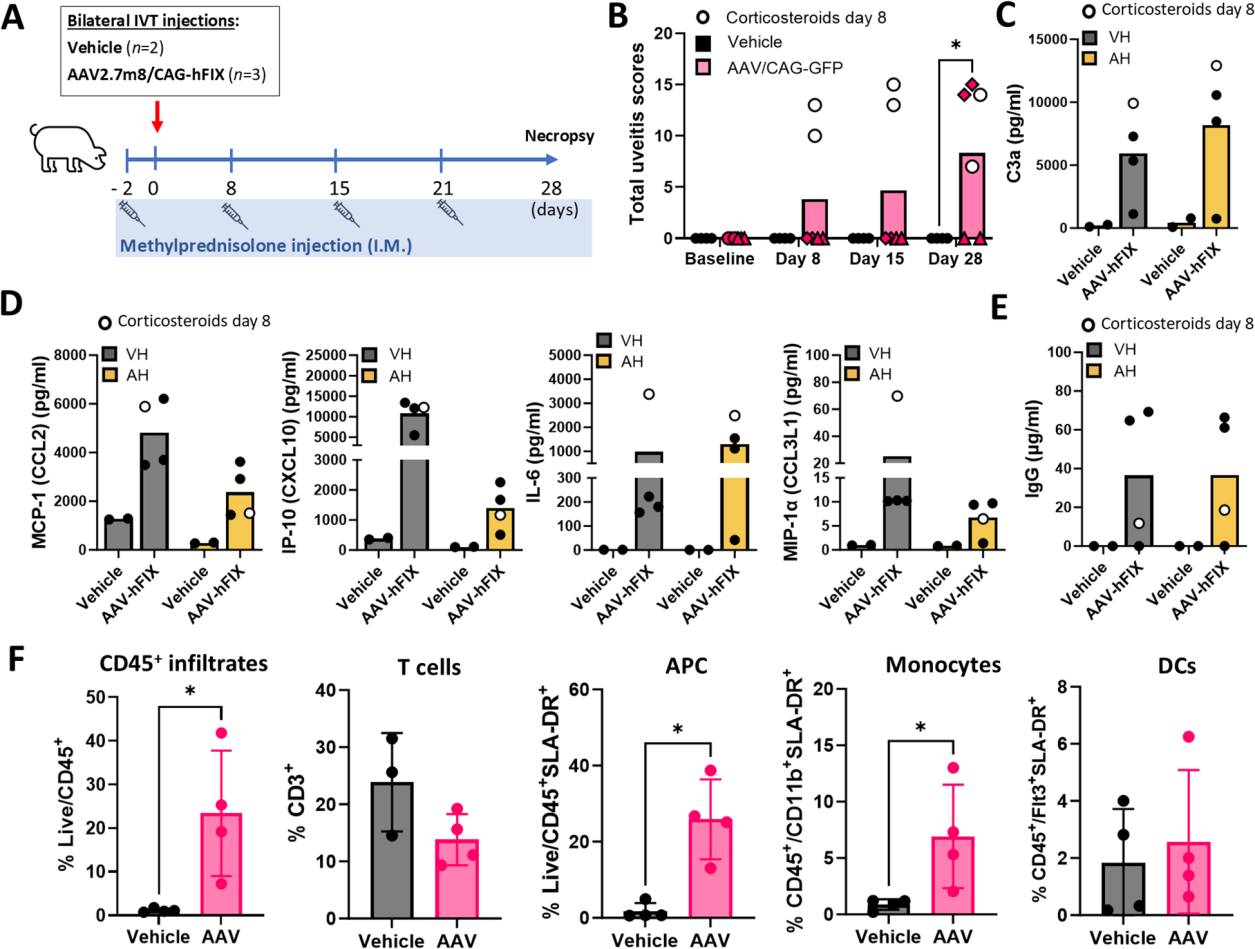
Preliminary assessment of intravitreal gene transfer in the pig shows early inflammation post-AAV injection. **(A)** Animals received intravitreal (IVT) injections of AAV2.7m8/CAG-hFIX (n = 3) at 5x1011 vg/eye or vehicle (n = 2) on day 0. Prophylactic methylprednisolone was administered intramuscularly (I.M.) from day -2 and weekly thereafter. Animals were sacrificed on day 28. **(B)** Total uveitis scores in AAV2.7m8/CAG-hFIX-injected eyes (n = 6) overtime, compared to vehicle control eyes (n = 4) at terminal timepoint. Bars represent mean uveitis score. Symbols show individual scores in each eye. Different symbols represent pairs of eyes belonging to the same animal. Open circles identify eyes from the animal that received corticosteroids on Day 8. Statistical significance was determined by two-way ANOVA with Bonferroni correction. *P = 0.0367. Analysis of **(C)** complement C3a, **(D)** cytokines and chemokines MCP-1, IP-10, IL-6 and MIP-1α and **(E)** anti-AAV2.7m8 IgG in aqueous (AH) and vitreous (VH) humors at terminal timepoint. For C-E, data are represented as mean between 2 vehicle- and 4 AAV-injected eyes. **(F)** Flow cytometry analysis of the frequencies of CD45+ cells, T cells, antigen presenting cells (APC), monocytes and dendritic cells (DCs) in total ocular homogenates. Data are shown as mean ± SD between 4 vehicle- and 4 AAV-injected eyes. Statistical significance was determined by Mann-Whitney t-test. In all cases, *p = 0.0286.

To verify the induction of GTAU upon IVT AAV2.7m8 injection in pigs, we first conducted a small, 28-day study in female Yucatan minipigs (3–5 months old). Animals, pre-selected for the lack of AAV2.7m8 neutralizing antibodies, received bilateral IVT injections of AAV2.7m8 (n = 3, 5×10^11^ vg/eye) encoding human coagulation factor IX (hFIX), used here as a model transgene, or vehicle (n = 2). All animals received systemic prophylactic methylprednisolone (5 mg/kg, maximum tolerated dose in this species), 2 days prior to AAV injection and weekly thereafter (**Figure 1A**).

We evaluated inflammation through ocular examinations and semi-quantitative uveitis scoring used in veterinary medicine (38). Despite the use of systemic steroids, one AAV-injected animal developed uveitis in both eyes one week post-AAV delivery and was administered topical corticosteroids once on day 8 post-injection. In this animal, uveitis scores continued to increase in both eyes over the 4-week period, despite the addition of topical steroids (**Figure 1B**). Of the other 2 animals, one did not develop uveitis in either eye during the follow up, and the other exhibited clinically apparent uveitis in both eyes (**Figure 1B**).

Additionally, we collected aqueous humor (AH) and vitreous humor (VH) samples at the terminal 4-week timepoint and assessed levels of secreted pro-inflammatory factors in 2 out of 4 vehicle-injected and 4 out of 6 AAV-injected eyes; 1 eye with a uveitis score of 0, and 3 eyes with uveitis scores of 14-15 (2 of 3 eyes from the same animal). Our analysis demonstrated activation of the complement pathway, which is known to be induced by systemic AAV gene transfer (39), as evidenced by elevated C3a levels relative to the vehicle-injected group (**Figure 1C**). The levels of MCP-1, IP-10, IL-6 and MIP-1α were also robustly elevated in porcine ocular humors (**Figure 1D**). In general, complement and cytokine/chemokine elevations were more pronounced in vitreous samples, and were apparent even in the animal that received additional topical steroids. As expected from previous reports (11), most animals developed anti-AAV2.7m8 IgG antibodies, which were detectable in both VH and AH samples independent of the uveitis scores (**Figure 1E**). Finally, flow cytometry analysis showed ocular infiltration of peripheral CD45^+^ cells including antigen presenting cells and monocytes (**Figure 1F**). Altogether, this preliminary study helped establish a dose of AAV2.7m8 capable of inducing GTAU in pigs and identify markers of inflammation to support further investigation of IVT AAV-induced IOI in this species.

### IVT AAV delivery transduced multiple cell types in pig retina with the highest transgene expression in GCL

**Figure 2:**
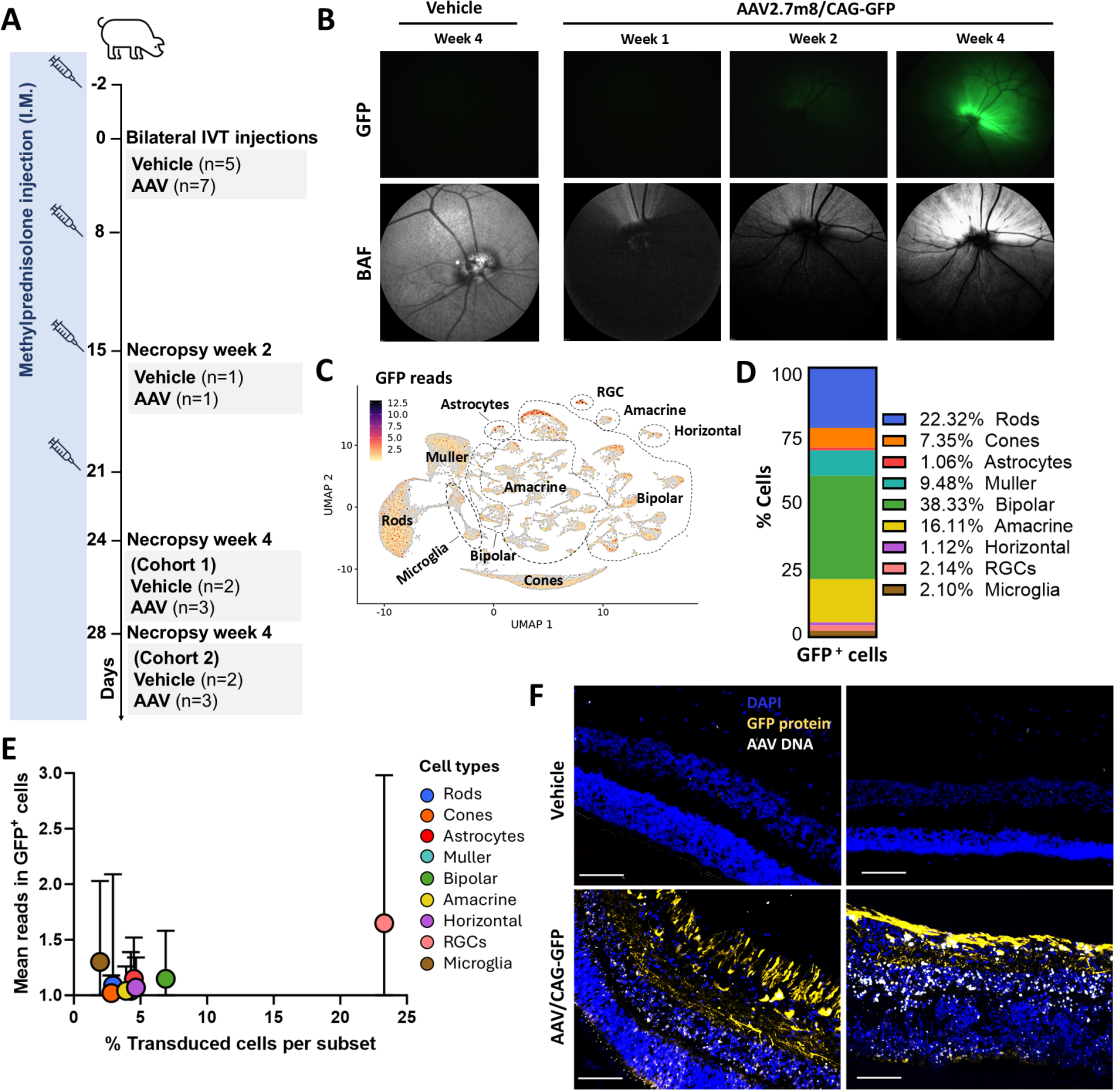
Intravitreally dosed AAV2.7m8 distributed across the pig retina and induced the highest transgene expression in GCL. **(A)** Animals received bilateral intravitreal (IVT) injections of AAV2.7m8/CAG-GFP (n = 7) at 5x1011 vg/eye or vehicle (n = 5) on day 0. Prophylactic methylprednisolone was administered intramuscularly (I.M.) from day -2 and weekly thereafter. Animals were sacrificed on days 15 (week 2), 24 (week 4 cohort 1) or 28 (week 4 cohort 2) as indicated. **(B)** Representative fundus images of GFP fluorescence (top) and confocal scanning laser ophthalmoscopy with blue autofluorescence (BAF) (bottom) in vehicle- and AAV-injected eyes at different timepoints post-injection. **(C)** UMAP of GFP mRNA distribution. **(D)** Stacked barplot showing the cell type frequencies among GFP+ nuclei. **(E)** Quantification of the percentage of GFP mRNA+ cells and mean GFP reads per cell population in AAV-injected retinas on week 4 presented as mean ± SD per cell population. **(F)** Representative IF images of GFP protein expression (yellow) and AAV vector genomes by RNAscope using probes against the CAG promoter (white), in retina sections from two different vehicle- and AAV-injected animals, 4 weeks post-injection. Sale bar 100μM.

To explore the underlying mechanisms of GTAU further in a follow-up study, we dosed pigs bilaterally with an AAV2.7m8 vector encoding GFP, which enabled periodic in-life detection of transgene expression in transduced cells. Animals received IVT injections of AAV2.7m8/CAG-GFP (5×10^11^ vg/eye, n = 7) or vehicle (n = 5), together with weekly systemic methylprednisolone (**Figure 2A**). In-live fundus and optical coherence tomography (OCT) imaging with blue autofluorescence (BAF) detected GFP signal beginning 2 weeks post-AAV injection and increasing up to termination at week 4 (**Figure 2B**). As expected for IVT delivery, fluorescence microscopy of retina sections prepared at week 4 showed the strongest GFP fluorescence in the GCL of the inner retina, colocalized with the RGC marker Brn3a (70.65% ± 7.78 of Brn3a^+^ cells were GFP ^+^) (**Supplementary Figures 1A, B**). Of note, quantification of the percentage of Brn3a^+^ cells revealed similar RGC numbers in vehicle- and AAV-injected retinas (**Supplementary Figure 1C**), suggesting no major loss of RGCs despite the high transgene expression in this cell type.

Next, we performed single-nucleus RNA sequencing (snRNAseq) analysis to assess the distribution of transgene expression among retinal cell subsets, with higher specificity compared to GFP fluorescence measurement. To this end, nuclei from dissected retinas were obtained from vehicle- or AAV-injected animals, 2 or 4 weeks post-AAV injection (n = 2 per condition). Based on the expression of 48 phenotypic markers (**Supplementary Figure 2A**), we identified subsets of cones, rods, astrocytes, RGCs, microglia, bipolar, amacrine, and horizontal cells (**Supplementary Figures 2B, C**). As expected for the AAV2.7m8 capsid and the ubiquitous CAG promoter, GFP transcripts could be detected at various levels in all identified cell populations (**Figures 2C–E**). Analysis of GFP^+^ nuclei demonstrated that the major cell populations containing at least 1 transcript per cell corresponded to bipolar (38.3% of GFP^+^ nuclei), rods (22.3%) and amacrine cells (16.1%) (**Figures 2C, D**), correlating well with the relative abundance of these cells in the retina. On the other hand, analysis of cell transduction within each cell population identified the highest percentage of GFP mRNA^+^ cells and GFP reads per cell in RGCs (**Figure 2E**), consistent with the high detection of GFP fluorescence in this cell type. Finally, we performed RNAscope with probes against the CAG promoter to study AAV vector genome distribution throughout the retina (**Figure 2F**). RNAscope confirmed that AAV2.7m8 genomes were distributed across all analyzed retinal layers.

In summary, the observed pattern of transgene expression in the porcine retina aligns with findings in other preclinical models of IVT AAV2.7m8 gene transfer.

### Intravitreally dosed AAV2.7m8 activates robust antiviral and DNA damage responses in porcine retinas

**Figure 3:**
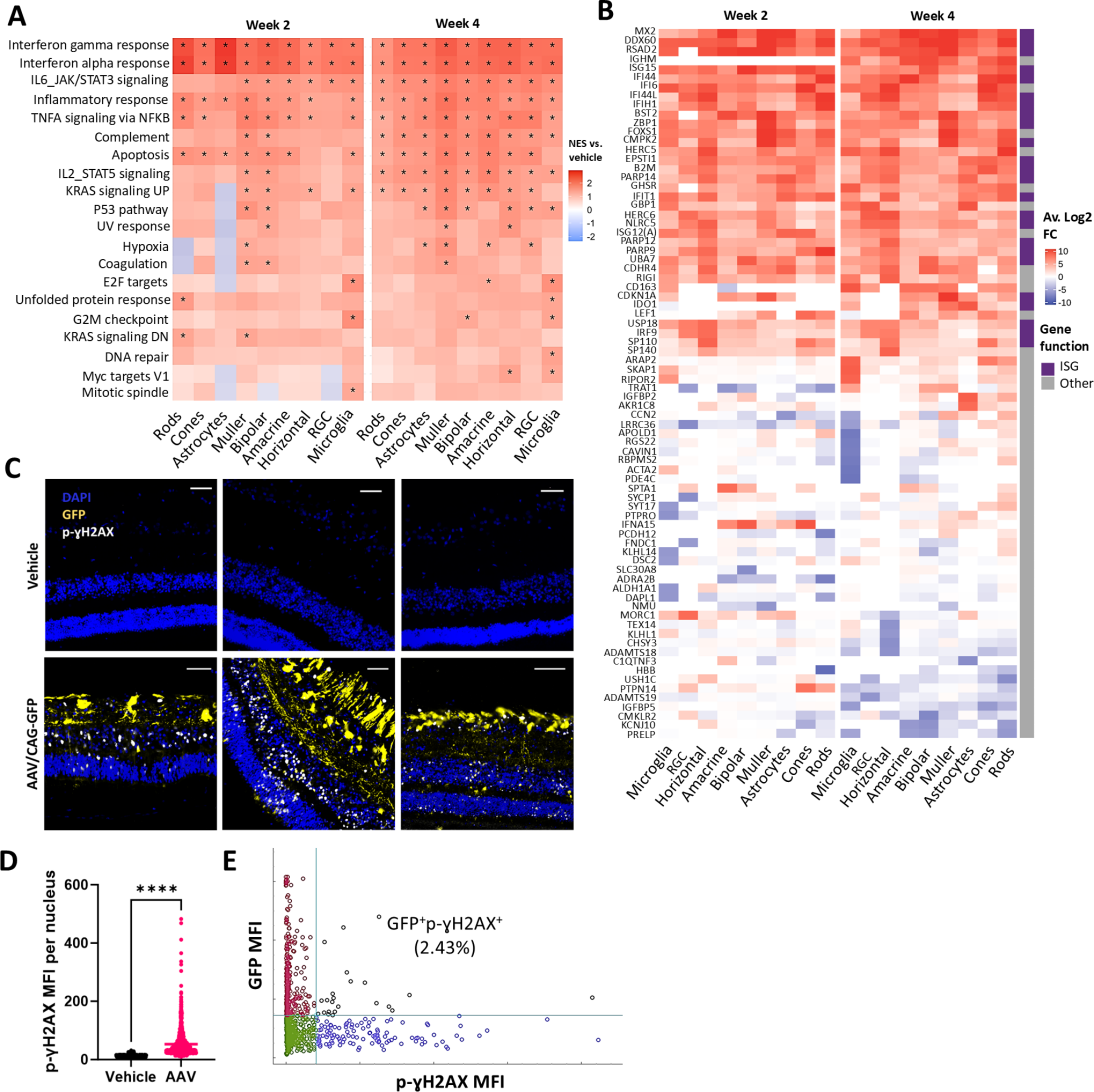
Intravitreally dosed AAV2.7m8 activates DNA damage and robust antiviral responses in pig retinas. **(A)** Heatmaps showing enriched GSEA terms in retinas from AAV-injected animals collected 2- and 4-weeks post-injection, against the Hallmark gene set (Molecular Signatures Database). Each column represents the average between 2 eyes. GSEA was performed on logFC fold changes in gene expression compared to vehicle controls (NES, normalized enrichment score; *, adjusted p <0.05). **(B)** Heatmaps showing top upregulated and downregulated genes in retinas from AAV-injected animals at the indicated timepoints and their contribution to IFN signaling according to the Hallmark gene set. ISG, interferon stimulated gene. **(C)** Representative IF images of p-ɣH2AX+ foci and GFP expression in retina sections from different vehicle and AAV-injected animals, 4 weeks post-injection. Scale bar 200μM. **(D)** Quantification of nuclear p-ɣH2AX mean fluorescence intensity (MFI) per cell. Dots represent MFI values per individual DAPI+ nucleus, pooled from 3 vehicle- and 5 AAV-injected animals, 2 ROIs per animal. Statistical significance was determined by Welch’s t-test. **(E)** Representative scatter plot of GFP and p-ɣH2AX colocalization analysis from one representative ROI, generated with MACS IQ View Software. **** symbol corresponds to the P < 0.0001.

Next, we evaluated the signaling pathways activated by IVT AAV injection as prospective targets for inflammation prevention. We conducted Gene Set Enrichment Analysis (GSEA) on the snRNAseq dataset at 2 and 4 weeks post-AAV injection, using bulk cell populations comprising both GFP^+^ and GFP^-^ cells. GSEA pathway analysis revealed that AAV injections led to significant upregulation of inflammation and cell toxicity pathways, including IFN, IL-6, TNFα, apoptosis, and complement, in all analyzed retinal populations (**Figure 3A**). Changes in gene expression were exacerbated at 4 weeks post-AAV injection compared to 2 weeks. When focusing on the top upregulated genes, we observed that these were generally common across cell clusters and mainly corresponded to interferon-stimulated genes (ISGs) (**Figure 3B**). In contrast, no consistent pattern was identified among downregulated genes across cell populations.

Notably, transcriptional changes in the p53 pathway, a sign of DNA damage response (DDR), were induced at 2 weeks post-AAV injection in Müller and bipolar cells, whose nuclei reside in the inner nuclear layer (INL), and extended to astrocytes, horizontal cells, RGCs, and microglia by week 4 (**Figure 3A**). We have previously reported a link between p53 activation and inflammation in the central nervous system (CNS) *in vitro* and *in vivo* upon AAV transduction (40). To confirm functional induction of DDR, we stained retinal sections for the presence of phosphorylated γH2AX (p-γH2AX) DNA damage foci. Results shown in **Figures 3C** and **D** confirmed significant p-γH2AX staining across retinal layers by week 4. Of note, high GFP fluorescence did not correlate with high γH2AX foci formation (**Figure 3E**). In line with this observation, snRNAseq analysis did not detect a correlation between GFP reads and the expression of the p53-dependent gene CDKN1A (**Supplementary Figure 3**). Both results suggested that DDR was not related to high levels of transgene expression. Additionally, p-γH2AX foci were also observed in pig retinas transduced with AAV2.7m8-hFIX (**Supplementary Figure 4**), excluding the possibility that DDR induction resulted from GFP toxicity.

Overall, our results highlight the induction of IFN, DDR and cell stress in cells internalizing the AAV vector genome independent of transgene expression levels, suggesting variable susceptibility of different cell populations to the presence of vector genomes.

### Kinetic assessment shows early inflammation accompanied by complement activation and chemokine accumulation in ocular humors

**Figure 4:**
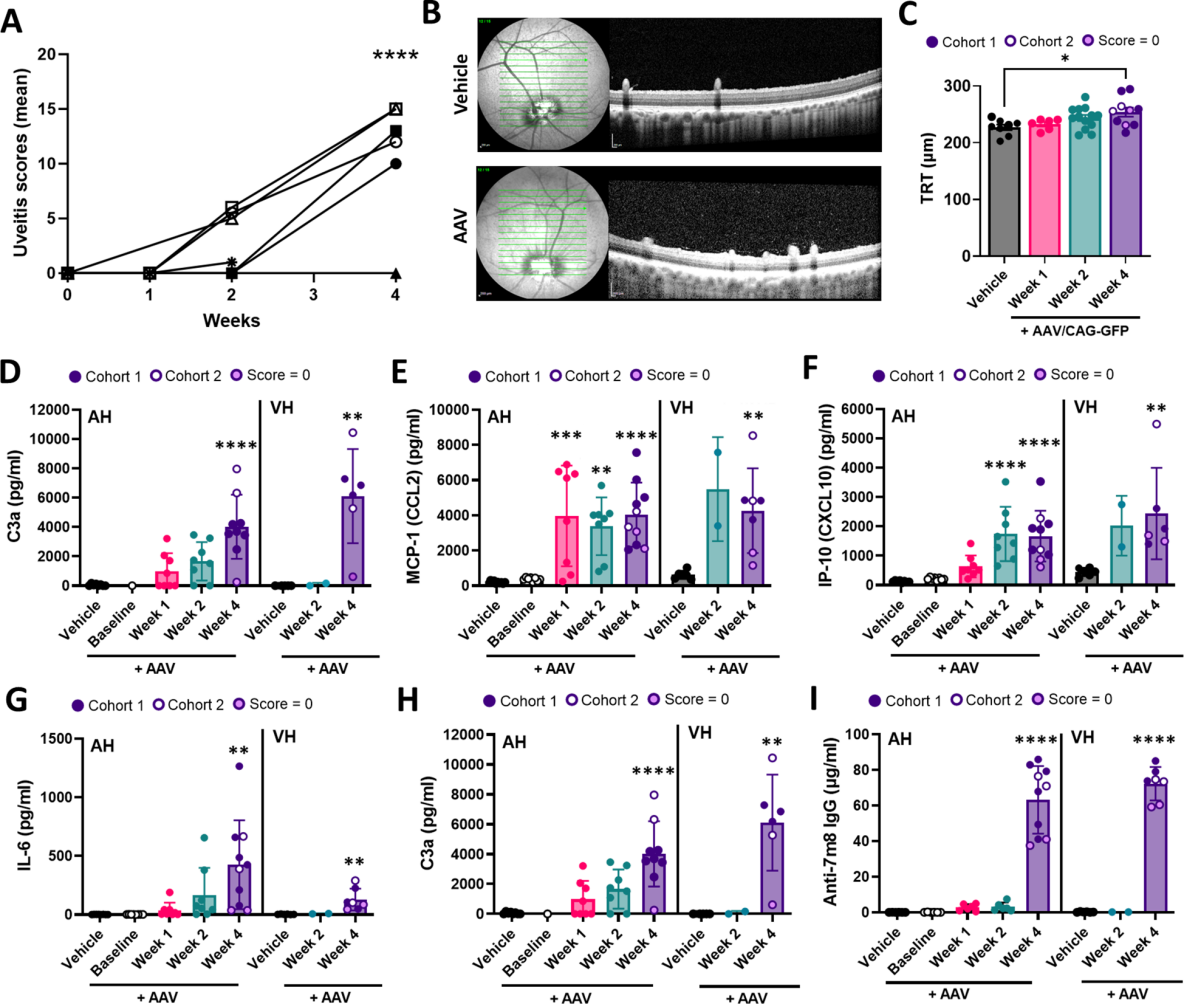
Kinetic assessment shows early inflammation accompanied by complement activation, chemokine accumulation and anti-AAV IgG development in ocular humors. **(A)** Total uveitis scores in AAV/GFP-injected animals over time. Data correspond to the mean between both eyes from the same animal from 7 AAV-injected animals. Statistical significance against vehicle week 4 was determined by one-way ANOVA with Tukey correction. **** P < 0.0001 vs. baseline (week 0). **(B)** Representative OCT showing horizontal scans from vehicle- and AAV-injected retinas at terminal timepoint week 4. **(C)** Quantification of total retinal thickness (TRT) derived from horizontal retinal scans over time. Data correspond to individual eyes from 4 vehicle controls (week 4) and 7 AAV-injected animals over time, and are represented as mean ± SD. Statistical significance was determined by one-way ANOVA with Tukey correction. *P = 0.0383. Analysis of **(D)** complement factor C3a, **(E)** MCP-1, **(F)** IP-10, **(G)** IL-6 and **(H)** MIP-1α and **(I)** anti-AAV2.7m8 IgG in AH and VH at the indicated timepoints. For C-I, eyes with a uveitis score of 0 belong to cohort 2 and are highlighted in light purple. For C-H, data are represented as mean ± SD between individual eyes from 5 vehicle- and 7 AAV/GFP-injected animals. For detailed N refer to supplementary table 5. For AH samples, statistical significance between all timepoints vs. vehicle was determined by one-way ANOVA with Tukey correction. For VH samples, statistical significance between week 4 group and vehicle was determined by Mann-Whitney t-test. *P < 0.05, **p < 0.01, p < 0.001, *** p < 0.0001 vs. vehicle.

Transcriptomic alterations aligned with a time-dependent elevation in uveitis scores, which were significantly increased at week 4 in all but one animal (**Figure 4A**). Both eyes from each animal consistently displayed comparable scores (**Supplementary Tables 1**–**4**). Additionally, OCT scans of pig eyes revealed cellular infiltration within the vitreous humor (**Figure 4B**) and a modest rise in total retinal thickness (TRT) at week 4 relative to vehicle controls (**Figure 4C**), thereby confirming the occurrence of ocular inflammation.

Next, we measured additional immune readouts in ocular humor samples (**Supplementary Table 5**). In agreement with SnRNAseq analysis showing complement pathway upregulation in bipolar and Müller cells at week 2, and in all analyzed retinal cell subsets at 4 weeks post-injection (**Figure 3A**), we detected evidence of complement activation in ocular humors (**Figure 4D**). C3a was elevated in AH as early as week 1 with levels increasing through week 4and in VH at week 4. (**Figure 4D**). Similarly, MCP-1 and IP-10 chemokines were elevated significantly as early as week 1 in AH and week 2 in VH with increased expression persisting through week 4 (**Figures 4E, F**). Interestingly, the induction of these pro-inflammatory biomarkers preceded detection of GFP fluorescence in the retina and the onset of elevated inflammation scores on ocular examination. IL-6 and MIP-1α were detectable at low levels at weeks 1 and 2 in AH and increased significantly in both AH and VH at the terminal timepoint (**Figures 4G, H**), potentially indicating delayed response kinetics compared to MCP-1 and IP-10. Overall, levels of C3a and pro-inflammatory factors tended to be lower in eyes with uveitis score of zero. Consistent with the findings from the initial porcine study, anti-AAV IgG levels were comparable across all injected eyes, including those that exhibited no clinical signs of uveitis (**Figure 4I**).

In summary, these results, consistent with the strong anti-viral response indicated by snRNAseq analysis, validate the induction of cytokines and chemokines and the activation of complement in GTAU.

### IVT AAV administration results in pro-inflammatory microglia accumulation in pig retinas

**Figure 5:**
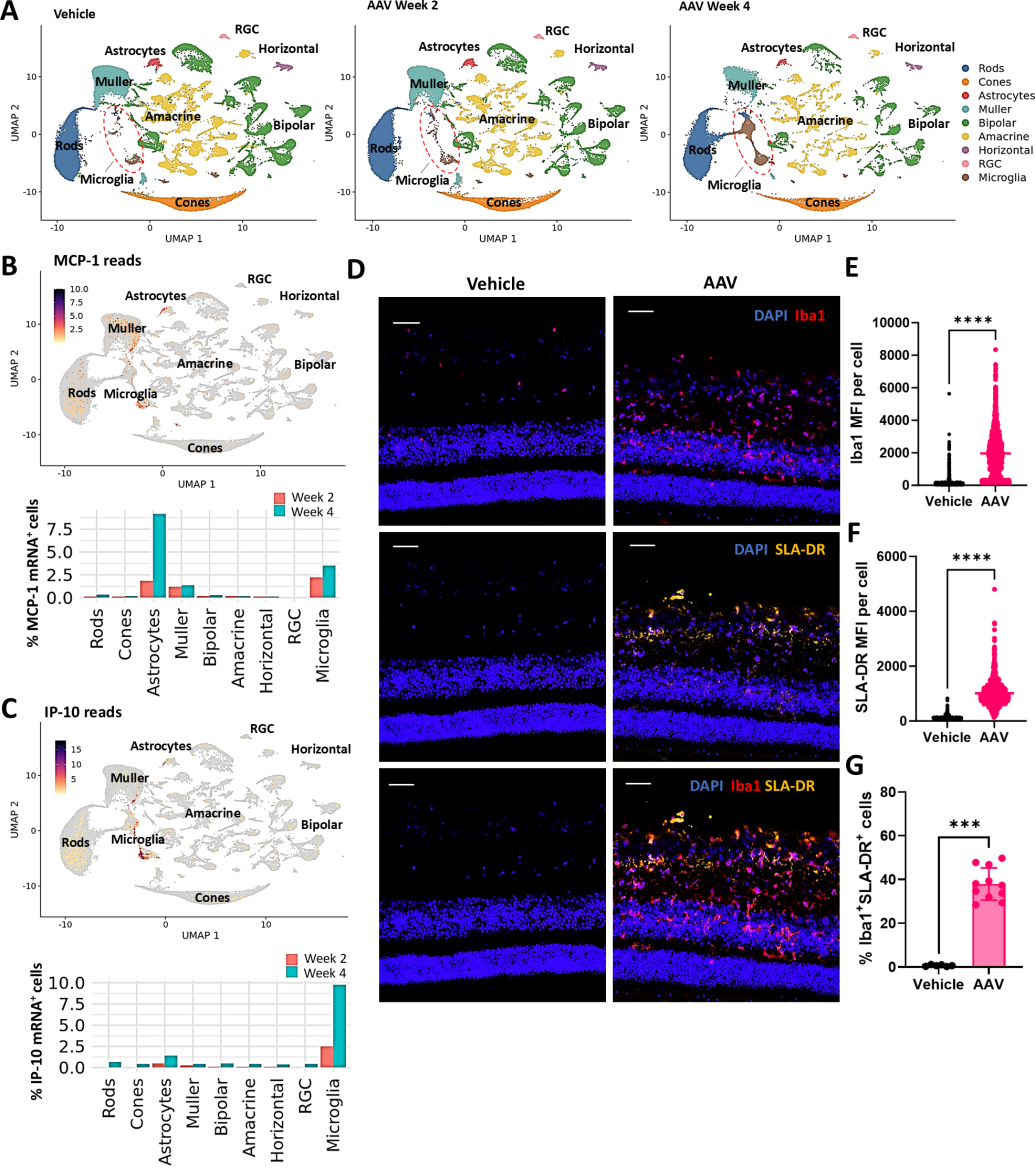
AAV-mediated retinal transduction leads to microglia activation. **(A)** UMAP plots showing cell populations identified in retinas from vehicle- and AAV-injected animals generated with Seurat. Plots show clustered results from 2 eyes per condition. UMAP of gene expression distribution and quantification of the percentage of **(B)** MCP-1 and **(C)** IP-10 mRNA-positive cells per cluster in AAV-injected retinas. **(D)** Representative IF images of Iba1 and SLA-DR staining in retina sections. Scale bar 200μM. Quantification of **(E)** Iba1 and **(F)** SLA-DR cell stainings in retina sections from vehicle- and AAV-injected animals 4 weeks post-injection. For E and F, individual dots represent mean fluorescence intensity (MFI) values per DAPI^+^ cell, pooled from 3 vehicle- and 5 AAV-injected animals, 2 ROIs per animal. Statistical significance was determined by Welch’s t-test. **** P < 0.0001. **(G)** Quantification of the percentage of Iba1^+^SLA-DR^+^ double positive cells. Data are shown as mean ± SD between individual ROIs from 3 vehicle- and 5 AAV-injected animals, 2 ROIs per animal. Statistical significance was determined by Mann-Whitney t-test. ***P = 0.0002.

SnRNAseq analysis of retinal cell clusters from AAV-GFP-injected eyes suggested an increase in microglia number over time (**Figure 5A**). Microglia appeared to exhibit a pro-inflammatory profile, as evidenced by the overexpression of MCP-1 and IP-10 encoding genes in this cell subset (**Figures 5B, C**). Immunofluorescence staining confirmed the increase in Iba1^+^ positive cells in retina sections from AAV-injected pigs, particularly in the GCL and INL (**Figures 5D, E**). Moreover, Iba1^+^ cells from injected animals showed upregulated Swine Leukocyte Antigen (SLA) major histocompatibility complex (MHC) class II DR molecules (SLA-DR) (**Figures 5D, F, G**), consistent with microglia activation and in line with previous studies of subretinal and IVT AAV delivery in animal models (13, 34).

Astrocytes also exhibited elevated MCP-1 transcriptional expression, potentially indicative of astrogliosis (**Figure 5B**). On the other hand, immunofluorescence imaging and quantification of Glial Fibrillary Acidic Protein (GFAP), expressed by both astrocytes and Müller glia, did not demonstrate significant differences between AAV-treated animals and vehicle controls. Morphological features consistent with gliosis were observed in only a few sections from a single AAV-injected animal with a uveitis score of 13 (**Supplementary Figure 5**). Increased frequency of the Iba1^+^SLA-DR^+^ microglia with no changes in GFAP staining was also observed consistently upon AAV2.7m8/hFIX administration in pigs (**Supplementary Figure 6**).

Overall, these results indicate that microglia play a critical role in the immune response to AAV administered via intravitreal injection in pigs, regardless of the transgene expressed.

### Integrin blockade prevents IOI following IVT AAV2.7m8 vector dosing in mice

Our investigations in pigs, along with previous studies in mice, have shown the occurrence of peripheral infiltrates following IVT AAV injection (30, 32–34). Given the cost-effectiveness of mice as experimental models for mechanistic research, we performed an exploratory study in this species to assess the role of peripheral infiltrates in AAV-induced IOI. To that aim, mice were dosed with IVT AAV2.7m8 concomitant with anti-LFA-1 and anti-VLA-4 neutralizing antibodies, which have been shown to inhibit leukocyte trafficking and extravasation and reduce inflammation in autoimmune conditions (41) as well as uveitis models (42).

**Figure 6:**
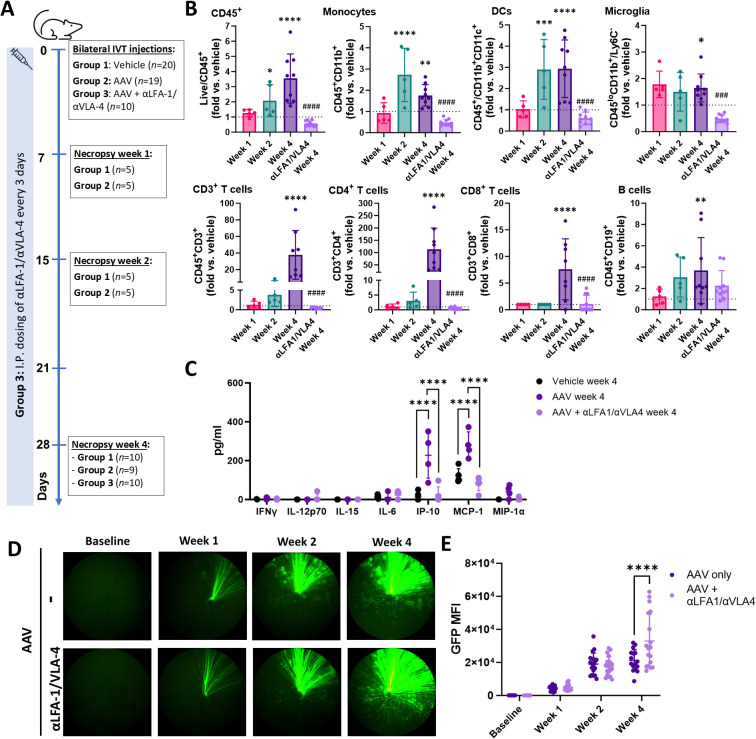
LFA-1/VLA-4 neutralization prevents IOI after intravitreal AAV vector dosing in mice. **(A)** Study design. Female C57Bl/6J mice received intravitreal (IVT) bilateral injections of AAV2.7m8/CAG-GFP (1x10^9^ vg/eye) or vehicle control on day 0 and sacrificed at different timepoints as indicated in the scheme. One cohort of AAV-injected mice received concomitant intraperitoneal (I.P.) injections of anti-LFA-1/VLA-4 antibodies on day 0, and every 3 days until termination (week 4). **(B)** FACS analysis of ocular infiltrates in ocular homogenates. Both eyes from from the same animal were pooled for analysis. Data are shown as mean ± SD between n = 5 week 1, n = 5 week 2, n = 9 week 4 and n = 10 αLFA-1/VLA-4-treated week 4 animals, and represented as fold change between AAV-injected animals relative to vehicle controls. The dashed line represents values in vehicle controls, set at 1. Statistical significance for each timepoint was determined respect to its own vehicle control by one-way ANOVA with Fisher’s LSD test (n = 5 for vehicles week 1 and 2, n = 10 vehicle week 4). *P < 0.05, **p < 0.01, ***p < 0.001, ****p < 0.0001 vs. vehicle; ### p < 0.001, #### p < 0.0001 vs. AAV week 4. **(C)** Quantification of cytokines and chemokines in vitreous humors. Each dot corresponds to a pool between 4 eyes from 2 animals from the same treatment group. Data are shown as ± SD between pools (n = 5 for vehicle and αLFA-1/VLA-4-treated animals, n = 4 for AAV-treated only). Statistical significance was determined by two-way ANOVA with Tukey’s correction. ****P < 0.0001. **(D)** Representative fundus images of GFP expression over time. **(E)** Quantification of GFP mean fluorescence intensity (MFI) in mice injected with AAV only (n = 8) or with AAV + anti-LFA-1/anti-VLA-4 (n = 9). Data are shown as mean ± SD between individual eyes. Statistical significance was determined by two-way ANOVA with Bonferroni correction. *** P = 0.0005.

Female C57BL/6 mice received bilateral IVT injections of either vehicle or AAV2.7m8/CAG-GFP at 1×10^9^ vg/eye in the absence of prophylactic steroids, followed by flow cytometry of whole ocular homogenates at 1, 2, and 4 weeks post-injection to assess leukocyte infiltration. An additional group of mice also received intraperitoneal (IP) injections of anti-LFA-1/VLA-4 neutralizing antibodies on day 0, and every 3 days until termination (**Figure 6A**). Flow cytometry (gating strategy illustrated in **Supplementary Figure 7**) demonstrated a significant accumulation of CD45^+^ monocytes and dendritic cells as early as two weeks following AAV injection. This was accompanied by an increase in microglia numbers and lymphocyte infiltration at 4 weeks, consisting predominantly of CD4^+^ T cells, with fewer CD8^+^ T cells observed. (**Figure 6B**). LFA-1/VLA-4 blockade effectively prevented infiltration of all affected immune cell populations (**Figure 6B**). Cytokine analysis of vitreous humor samples collected 4 weeks following AAV injection demonstrated elevated IP-10 and MCP-1 levels in mice treated with AAV only, which were significantly reduced by LFA-1/VLA-4 blockade (**Figure 6C**).

Color fundus imaging revealed a progressive increase in GFP transgene expression from week 1 to week 4 (**Figure 6D**). Notably, semi-quantitative evaluation of mean fluorescence intensity (MFI) indicated higher transgene expression in the subset of animals treated with LFA-1/VLA-4 blockade compared to those receiving only the AAV vector (**Figure 6E**; **Supplementary Figure 8**). These findings suggest that peripheral immune cell infiltration plays a critical role in driving GTAU in mice and may influence transgene expression outcomes.

## Discussion

This study presents mechanistic insights into the induction of GTAU following IVT AAV2.7m8 administration in pigs that may facilitate the development of innovative immunomodulatory approaches addressing current challenges of IVT AAV vector delivery.

SnRNAseq and RNAscope analyses performed on pig retinas confirmed AAV2.7m8 vector distribution across all retinal layers, with the highest transgene expression detected in RGCs, both in terms of transcript levels and protein detection. These results suggest that the pig model is well aligned in this respect with other preclinical models of IVT AAV administration.

Our previous work in mice and human iPSC-derived neural cells revealed that AAV vectors triggered neuroinflammation dependent on activation of DDR (40). The retina contains several types of neuronal cells that are essential for vision such as photoreceptors, bipolar cells, horizontal cells, amacrine cells, and RGCs. These neurons work together to process visual information and transmit it to the brain. Similarly to our previous findings in neuronal cell populations (40), snRNAseq analysis revealed that Müller and bipolar cells upregulated p53 signaling early in response to IVT AAV2.7m8, coinciding with the presence of γH2AX^+^ DDR foci in the INL where the nuclei of these cells are found. Müller cells are known to exhibit DDR in response to various forms of retinal damage, which can impact their behavior and regenerative potential (43, 44). DDR involves the activation of cell cycle checkpoints, DNA repair mechanisms and apoptosis, especially if the DNA damage is severe or persistent (45). Consistent with these reports, our snRNAseq analysis in pig retina identified the upregulation of apoptosis genes in Müller and other transduced cells. Further studies would be needed to confirm whether DDR leads to apoptosis and cell death in this setting.

Another aspect of the DDR response is its potential effect on transgene expression. ATM kinases and the MRN complex (Mre11/Rad50/Nbs1), which act upstream of p53 during DNA damage and repair (46, 47), have been reported to interact with the AAV inverted terminal repeats (ITRs) and suppress AAV-derived transgene expression (48, 49). Accordingly, the high concentration of p-γH2AX foci in retinal layers lacking GFP fluorescence, but containing AAV vector, points towards a potential role of DDR in preventing efficient transgene expression in certain retinal cell subsets. Additionally, the absence of correlation between high levels of GFP or hFIX transgene expression and p-γH2AX foci localization suggest that DDR was not caused by potential exogenous transgene toxicity in retinal cells.

Our snRNAseq analyses also identified IFN-induced genes as being predominantly enriched in IVT AAV-dosed retinas, with significant ISG upregulation in all analyzed retinal cell subsets. ISG induction has previously been reported in mice upon IVT (32) and subretinal injections (50, 51). ISGs are known to play a role as viral restriction factors, with the potential to interfere with viral nuclear entry, uncoating or spreading in the tissue. Therefore, we hypothesize that strong IFN signaling may hinder efficient transduction and transgene expression. In that case, strategies to inhibit IFN signaling should be further tested to assess their potential in driving broader and more efficient retina transduction and reducing required vector doses. Interestingly, IFN response occurred in retinal cells regardless of transduction levels and transgene expression, highlighting the immunogenicity risks of non-specific vector delivery to off-target cells.

Another important element of the immune response to AAV vectors is the complement pathway. Complement activation in response to systemic AAV administration and related acute toxicities has been reported for numerous clinical and preclinical studies (39, 52–56). In addition, complement often becomes activated in ocular inflammatory conditions outside of the gene therapy context (57, 58). We present here the first evidence of complement activation upon IVT AAV administration. Analysis of pig ocular samples revealed transcriptional upregulation of genes involved in the complement pathway, as well as accumulation of C3 convertase product C3a in ocular humors as early as 1 week post-AAV injection, before the peak of transgene expression. Importantly, C3a accumulation was induced by both transgenes tested, hFIX and GFP. Additional assessment in preclinical models would be needed to establish whether complement inhibition could mitigate AAV-mediated ocular inflammation, as suggested by studies in NHPs in which AAV was administered systemically (56).

IVT AAV injection also triggered MCP-1 and IP-10 secretion in ocular humors in mice and pigs. Importantly, both chemokines are known mediators of uveitis (59). MCP-1 levels were significantly elevated in porcine AH by week 1 post-injection, whereas in VH increased MCP-1 and IP-10 levels could be detected by week 2. Increased IL-6 and MIP-1α levels occurred later, indicating either delayed kinetics relative to MCP-1 and IP-10, lower sensitivity of detection, activation of different cell-types, or influence of transgene expression on their induction. As a corollary to these findings, it would be of interest to test the utility of C3a, MCP-1 and IP-10 measurements in AH as early predictors of IOI following the IVT AAV dosing in clinical trials, particularly where a favorable benefit-risk profile exists. Additionally, MCP-1 is crucial for monocyte recruitment to the CNS, and its neutralization can prevent microglia recruitment in brain (60). Accordingly, we observed peripheral monocyte infiltration and increased microglia frequency in both mice and pigs. More comprehensive analysis in pigs indicated that IVT AAV administration led to a transgene-independent pro-inflammatory profile of retinal microglia, as evidenced by over-expression of MCP-1 and IP-10 encoding genes, upregulation of interferon signaling and elevated levels of activation markers, such as Iba1 and SLA-DR in these cells.

In principle, pro-inflammatory cytokine signaling can result not only in the activation of resident immune cells, such as microglia, but also in the recruitment of peripheral immune cells to sites of inflammation. Our assessment of ocular infiltrates in mice reproduced previous reports demonstrating peripheral cell infiltration upon IVT AAV injection (32, 33). In particular, we observed significant T cell infiltration by week 4, with a high CD4^+^ T cell component. We did not observe significant T cell infiltration in pig retinas, although the absence T cell infiltration in our porcine model could be due to the effects of steroid treatment, implemented in pigs but not in mice (61, 62), or due to partial interspecies differences in the immune response to IVT-administered AAV. Leukocyte recruitment to tissues is mediated by ICAM-1 and VCAM-1 expression on retinal vascular endothelial cells and retinal pigment epithelium (RPE) cells. Both ICAM-1 and VCAM-1 are induced during intraocular inflammation (63). Dual VLA-4/LFA-1 blockade on circulating immune cells disrupts their interaction with ICAMs and VCAM-1 preventing leukocyte rolling, firm adhesion and extravasation (63). Notably, this treatment not only prevented peripheral infiltrates in murine eyes but also reduced pro-inflammatory chemokine levels in ocular humors, highlighting the role of peripheral infiltrates in mediating intraocular inflammation. We also observed a slight increase in GFP transgene expression in animals that received dual VLA-4/LFA-1 blockade compared to animals receiving only AAV vector. Of note, quantification of GFP transgene expression from color fundus images in mice is subject to technical limitations and to multiple sources of variation (e.g., injection quality, imaging artifacts, etc.). However, this observation was consistent with previous reports showing that reducing retinal inflammation may benefit transgene expression (30, 51). These observations suggest that further research into therapies designed to prevent activated immune cells from accessing ocular tissues may be warranted. Such interventions are currently utilized in the management of autoimmune diseases including multiple sclerosis and Crohn’s disease.

While inhibiting immune cell infiltration proved effective in preventing IOI in mice, this positive effect may be limited in larger species where inflammation appears to be more pronounced. Mice are valuable for studying ocular inflammation and screening therapeutics (64, 65), but interspecies differences in eye size, retinal structure, and immune composition may result in distinct immunogenicity outcomes, including IVT-AAV–induced inflammation in humans (66). Because pigs more closely resemble NHPs and humans in ocular anatomy and immune function, they serve as a relevant large-animal model to bridge murine findings to clinical settings (35). However, species-specific features of the porcine immune system warrant further validation of our findings.

Our work identified several immune mechanisms in response to IVT AAV-mediated gene transfer that could be further evaluated as targets for novel immunosuppressive approaches to improve safety of ocular gene therapies. These targets include IFN signaling, MCP-1 and IP-10 chemokines, DDR, the complement pathway, microglia activation and integrin-mediated peripheral immune cell infiltration. Although a single dominant trigger of AAV immunogenicity was not identified, modulation of key inflammatory pathways may mitigate vector-associated ocular inflammation. Intravitreal AAV administration in combination with systemic agents such as natalizumab—an approved therapy for relapsing–remitting multiple sclerosis with demonstrated efficacy and safety in reducing leukocyte trafficking—could potentially limit immune cell infiltration into inflamed ocular tissues. Complement activation also contributes to post-AAV inflammation, and intravitreal C3 inhibition with pegcetacoplan, approved for geographic atrophy, may serve as a complementary strategy to attenuate complement-mediated responses to gene therapy. Furthermore, future studies could explore the use of small-molecule STING inhibitors, currently in early-phase clinical development for interferonopathies and autoimmune diseases, as potential adjuncts to modulate AAV-induced innate immune responses. These approaches should first be evaluated in large animal models to establish safety and efficacy before clinical translation.

Limitations associated with IVT delivery of AAV vectors are well documented, including dose-dependent IOI, suboptimal effect of oral and topical corticosteroid treatments (27, 30), and inefficient transduction of outer retinal layers, in which vector genomes are present but transgene expression is low. Identification of strategies that prevent vector silencing could in theory allow for more efficient and broader retina transduction at reduced vector doses, which is a key factor in triggering inflammation. Additionally, the choice of AAV vector serotypes with cell-specific tropism and transgene expression would help minimize inflammatory signaling in off-target cells. Overall, a combination of such strategies may contribute to optimizing safety and efficacy of IVT AAV-mediated gene transfer in patients in which corticosteroids are contra-indicated or have limited efficacy.

## Data Availability

The data presented in the study in are deposited in the Gene Expression Omnibus (GEO) Repository, accession number GSE308982.
