# Supplementary material for: Intravitreal AAV vector delivery induces integrin-dependent ocular inflammation, complement activation, and antiviral and DNA damage responses
Source: Front Immunol. 2026 Apr 22;17:1799327. doi: 10.3389/fimmu.2026.1799327 (PMC13144134; doi:10.3389/fimmu.2026.1799327)
Supplement: Supplementary file 1 [file DataSheet1.pdf]

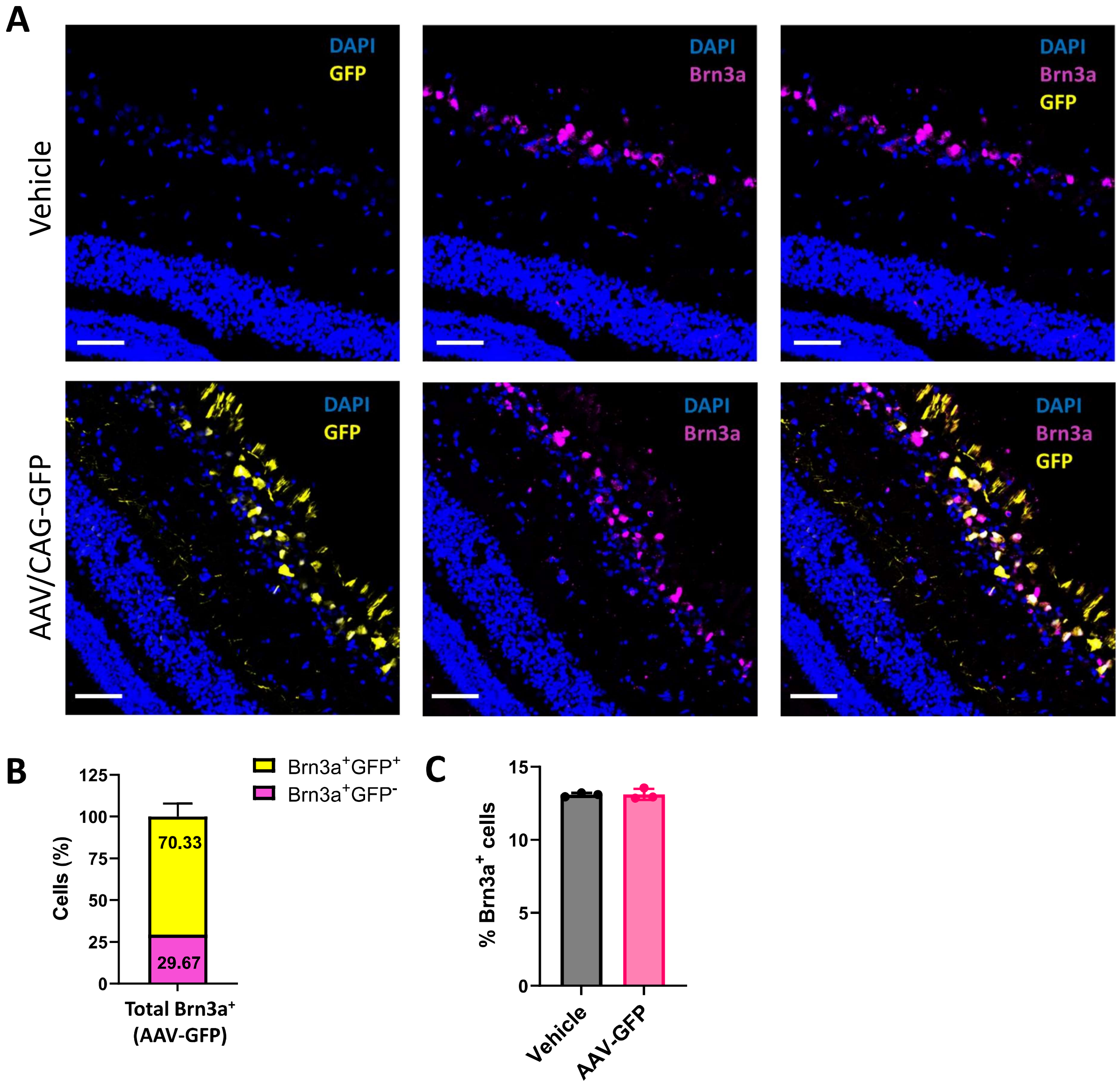

Supplementary figure 1. Distribution of transgene expression. A) Representative MACSima IF images of GFP fluorescence and Brn3a staining on retina sections from vehicle (top) and AAV/CAG-GFP (bottom) injected animals, 4 weeks post-injection. Scale bar 200µM. **B)** Quantification of the percent of GFP^+ and GFP^- cells gated on total $Brn3a^+$ cells using the MACS iQ View software. Data are shown as mean ± SD between 3 ROI from 1 AAV-injected animal. **C)** Quantification of the percent of $Brn3a^+$ cells on the total $DAPI^+$ cells. Data are shown as mean ± SD between 3 ROI from 1 vehicle- and 1 AAV-injected animal respectively.

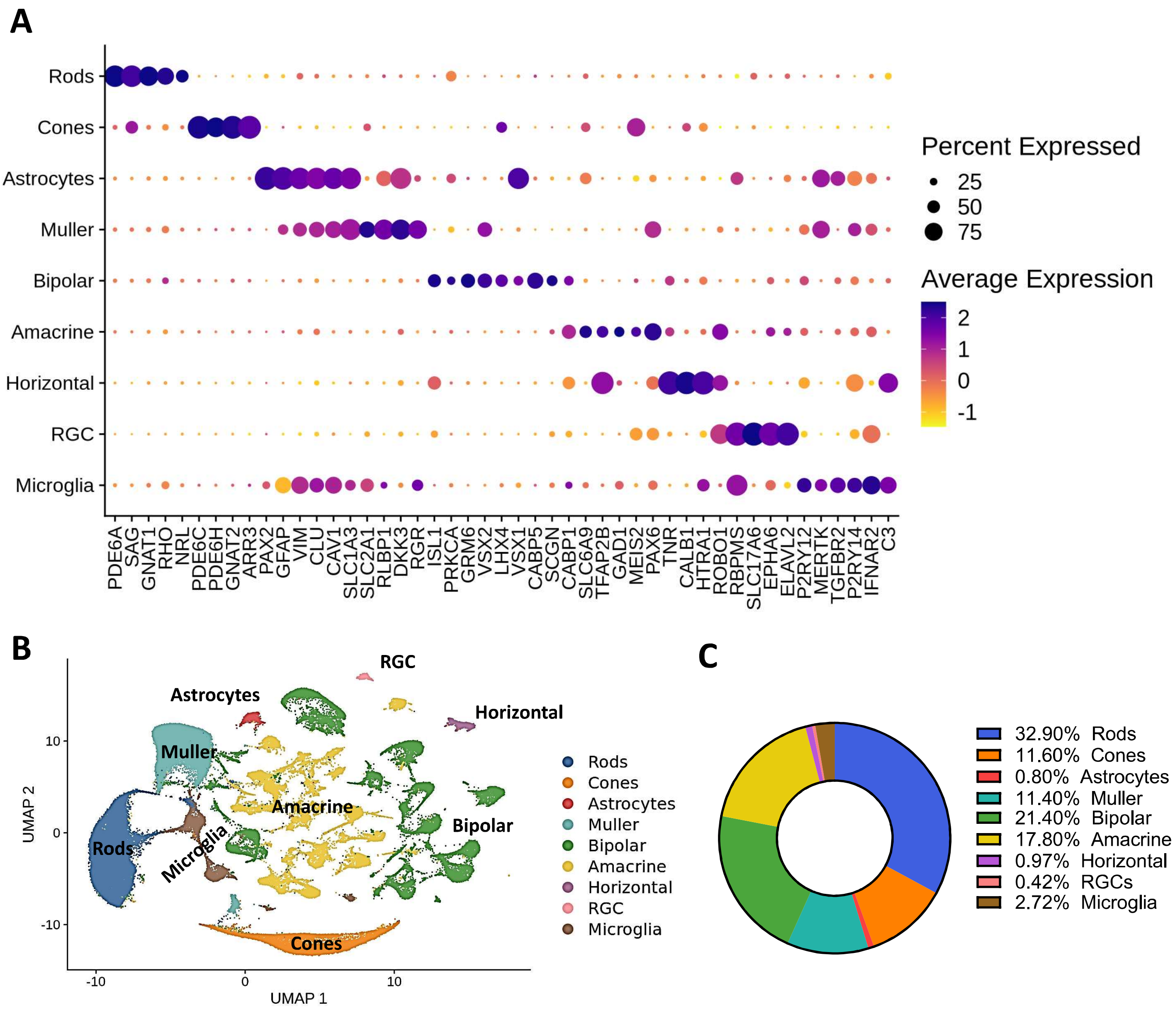

Supplementary figure 2. Identification of retinal cell subsets by snRNAseq. A) Dot plot representing the expression levels of selected retinal cell type marker genes to identify each cluster. The color of each dot represents the gene's expression level across the cluster (average expression), while the size of each dot corresponds to the percentage of cells in the cluster expressing the gene (percent expressed). **B)** UMAP plots of single-nuclei data generated with Seurat showing cell clusters identified in retina samples. Cells are plotted in two dimensions using the UMAP dimensionality reduction technique and annotated by cell type. **C)** Stacked plot representation of the frequencies for each cell population shown in B. A-C plots show clustered results from vehicle- and AAV-injected retinas obtained after 2- and 4-weeks post-injection, 2 eyes per condition. Plots were generated from clustered datasets between all eyes analyzed.

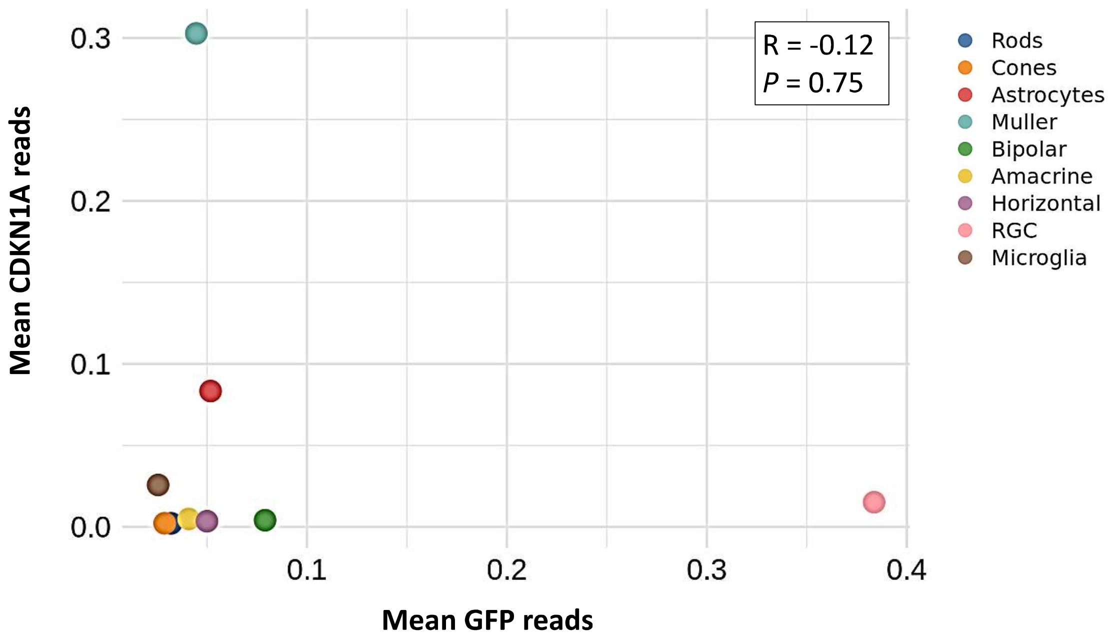

Supplementary figure 3. Correlation analysis between GFP and CDKN1A reads. Correlation analysis between the mean GFP reads and the mean CDKN1A reads per cell population measured 4 weeks post-AAV2.7m8/CAG-GFP injection.

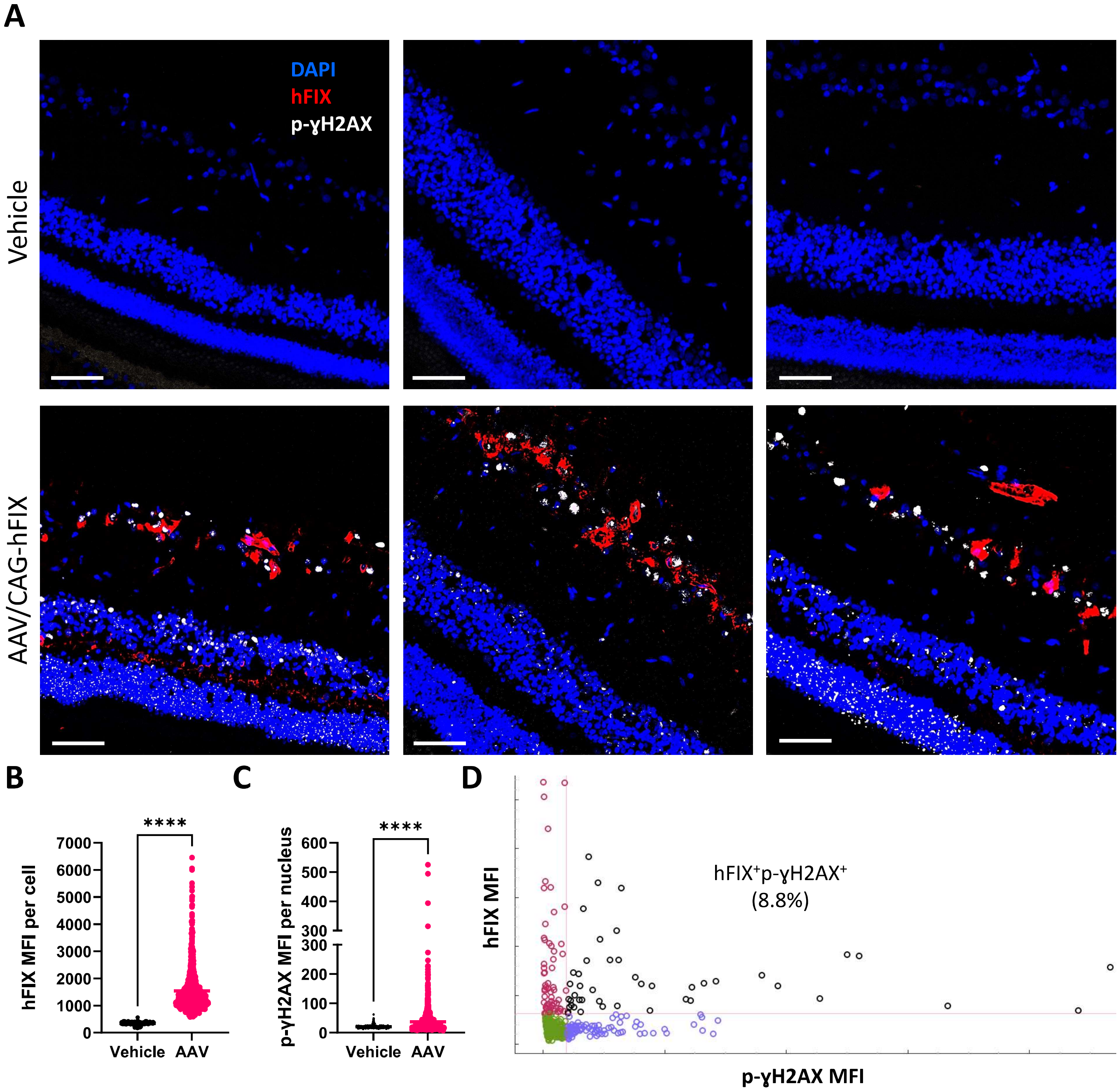

Supplementary figure 4. IVT-mediated retina transduction with AAV2.7m8 induces the formation of DNA damage foci. A) Representative MACSima IF images of hFIX and pospho-γH2AX stainings in vehicle (top) and AAV/CAG-hFIX (bottom) injected retinas 4 weeks post-injection. For each condition, ROIs correspond to different sections from the same animal. Scale bar 200μM. Quantification of intensity values for **B)** hFIX and **C)** p-ɣH2AX. Data correspond to mean fluorescence intensity (MFI) values per cell pooled from 3 ROIs from 1 vehicle and 1 AAV-injected animal respectively. Statistical significance was determined by Welch's t-test. **** $P < 0.0001$. **D)** Scatter plot of hFIX and p-ɣH2AX colocalization from one representative ROI, generated with MACS IQ view.

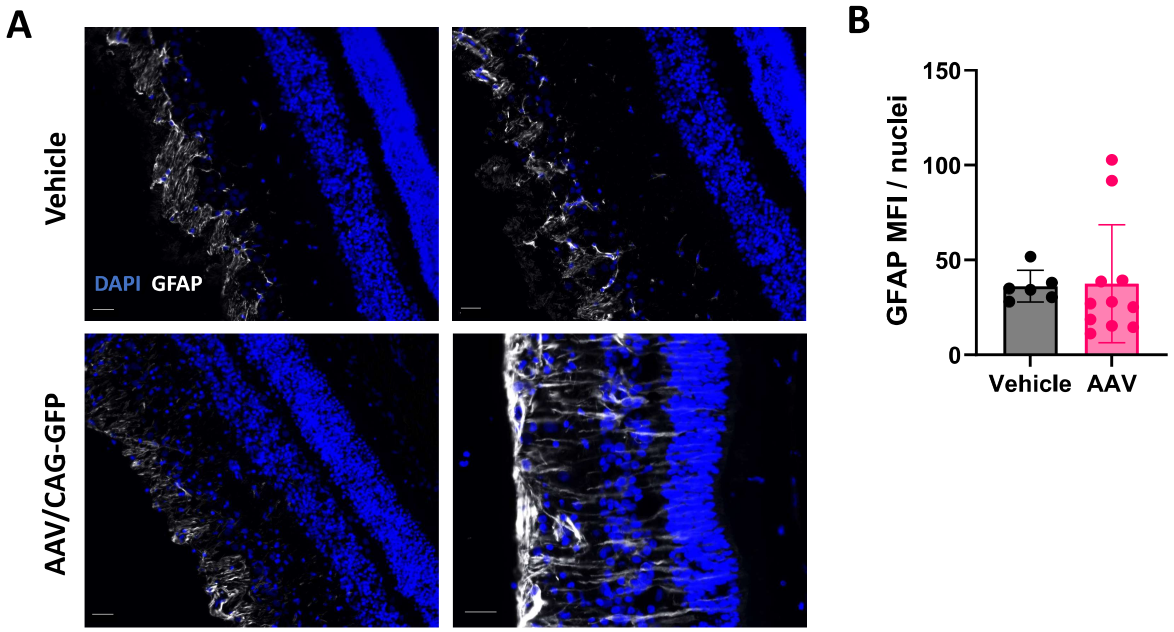

Supplementary figure 5. Assessment of astrogliosis. A) Representative IF images of GFAP staining in vehicle and AAV-injected animals 4 weeks post-injection. Scale bar 200µM. **B)** Quantification of GFAP mean fluorescence intensity (MFI). Data are shown as mean ± SD between individual ROIs from 3 vehicle and 5 AAV-injected animals, 2 ROIs per animal.

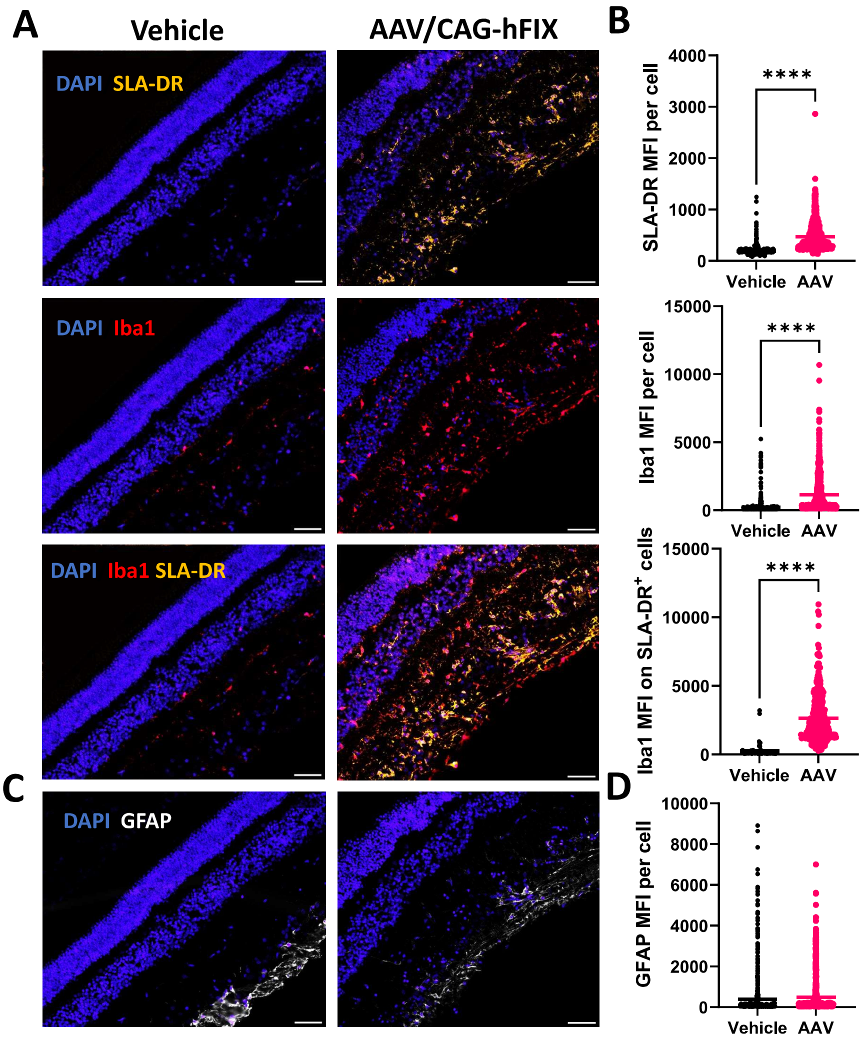

Supplementary figure 6. IVT-mediated retina transduction with hFIX transgene reproduces the microglia activation induced by GFP. A) Representative IF images of SLA-DR, IBA1 and GFAP stainings in vehicle- and AAV-injected animals 4 weeks post-injection. Scale bar 200µM. **B)** Quantification of intensity values for SLA-DR, Iba1, Iba1 in SLA-DR$^+$ cells. **C)** Representative IF images and **D)** quantification of GFAP staining. Data correspond to MFI values from single cells pooled from 1 vehicle and 1 AAV-injected animals, 2 ROIs per animal. Statistical significance was determined by Welch's t-test. **** $P < 0.0001$.

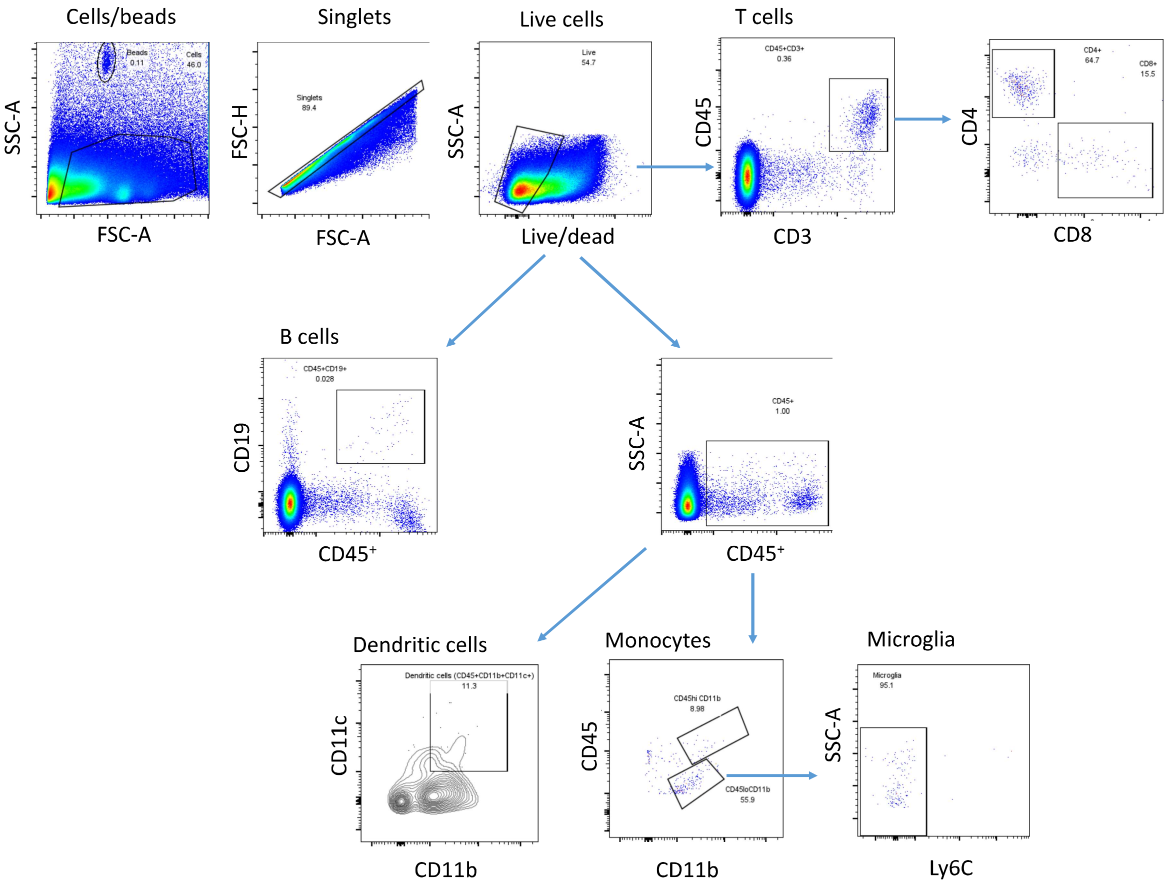

Supplementary figure 7. Immune response kinetics in mice. Gating strategy for flow cytometry analysis of murine eyes.

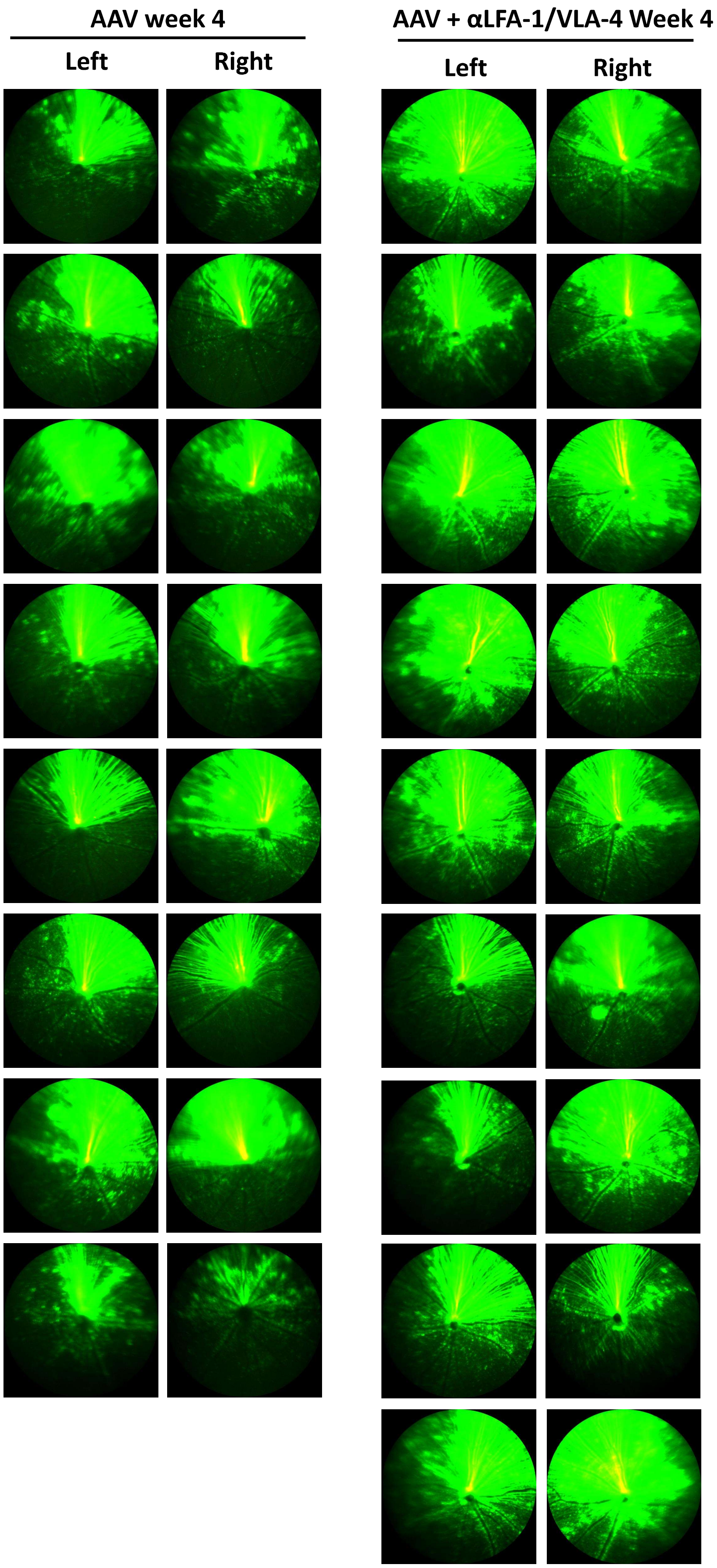

Supplementary figure 8. Fundus imaging of GFP fluorescence. Fundus images of individual eyes injected with AAV-GFP with (N=18) or without (N=16) concomitant treatment with αLFA-1/VLA-4 antibodies 4 weeks post-AAV injection.

Supplementary Table 1. Ocular evaluation of uveitis scores at baseline.

Baseline																					
			Anterior Segment											Posterior Segment							
			Conjunctiva			Cornea			Iris					Ocular Fundus							
Treatment	#Animal	Eye	Redness Congestion	Chemosis	Discharge	Opacity	Areas Involved	Cornea Pannus	Values	Aqueous Flare	Pupillary Light Reflex	Cellular Flare	Lens	Normal?	Vitreous Haze	Vitreous Cell	Optic Disc/Nerve	Retinal Vasculature	Retinal Pathology	Choroidal Pathology	**Total Score**
			(0-3)	(0-4)	(0-3)	(0-4)	(0-4)	(0-2)	(0-4)	(0-3)	(0-2)	(0-4)	(0-1)	Yes/No	(0-4)	(0-4)	(0-1)	(0-1)	(0-4)	(0-4)	
AAAV7m8-CAG-GFP	1	L	0	0	0	0	0	0	0	0	0	0	0	yes	0	0	0	0	0	0	**0**
		R	0	0	0	0	0	0	0	0	0	0	0	yes	0	0	0	0	0	0	**0**
	2	L	0	0	0	0	0	0	0	0	0	0	0	yes	0	0	0	0	0	0	**0**
		R	0	0	0	0	0	0	0	0	0	0	0	yes	0	0	0	0	0	0	**0**
	3	L	0	0	0	0	0	0	0	0	0	0	0	yes	0	0	0	0	0	0	**0**
		R	0	0	0	0	0	0	0	0	0	0	0	yes	0	0	0	0	0	0	**0**
	4	L	0	0	0	0	0	0	0	0	0	0	0	yes	0	0	0	0	0	0	**0**
		R	0	0	0	0	0	0	0	0	0	0	0	yes	0	0	0	0	0	0	**0**
	5	L	0	0	0	0	0	0	0	0	0	0	0	yes	0	0	0	0	0	0	**0**
		R	0	0	0	0	0	0	0	0	0	0	0	yes	0	0	0	0	0	0	**0**
	6	L	0	0	0	0	0	0	0	0	0	0	0	yes	0	0	0	0	0	0	**0**
		R	0	0	0	0	0	0	0	0	0	0	0	yes	0	0	0	0	0	0	**0**
	7	L	0	0	0	0	0	0	0	0	0	0	0	yes	0	0	0	0	0	0	**0**
		R	0	0	0	0	0	0	0	0	0	0	0	yes	0	0	0	0	0	0	**0**
Vehicle	8	L	0	0	0	0	0	0	0	0	0	0	0	yes	0	0	0	0	0	0	**0**
		R	0	0	0	0	0	0	0	0	0	0	0	yes	0	0	0	0	0	0	**0**
	9	L	0	0	0	0	0	0	0	0	0	0	0	yes	0	0	0	0	0	0	**0**
		R	0	0	0	0	0	0	0	0	0	0	0	yes	0	0	0	0	0	0	**0**
	10	L	0	0	0	0	0	0	0	0	0	0	0	yes	0	0	0	0	0	0	**0**
		R	0	0	0	0	0	0	0	0	0	0	0	yes	0	0	0	0	0	0	**0**
	11	L	0	0	0	0	0	0	0	0	0	0	0	yes	0	0	0	0	0	0	**0**
		R	0	0	0	0	0	0	0	0	0	0	0	yes	0	0	0	0	0	0	**0**
	12	L	0	0	0	0	0	0	0	0	0	0	0	yes	0	0	0	0	0	0	**0**
		R	0	0	0	0	0	0	0	0	0	0	0	yes	0	0	0	0	0	0	**0**

Supplementary Table 2. Ocular evaluation of uveitis scores at week 1 post-injection.

Week 1																					
			Anterior Segment											Posterior Segment							
			Conjunctiva			Cornea			Iris					Ocular Fundus							
Treatment	#Animal	Eye	Redness Congestion	Chemosis	Discharge	Opacity	Areas Involved	Cornea Pannus	Values	Aqueous Flare	Pupillary Light Reflex	Cellular Flare	Lens	Normal?	Vitreous Haze	Vitreous Cell	Optic Disc/Nerve	Retinal Vasculature	Retinal Pathology	Choroidal Pathology	**Total Score**
			(0-3)	(0-4)	(0-3)	(0-4)	(0-4)	(0-2)	(0-4)	(0-3)	(0-2)	(0-4)	(0-1)	Yes/No	(0-4)	(0-4)	(0-1)	(0-1)	(0-4)	(0-4)	
AAAAV7m8-CAG-GFP	1	L	-	-	-	-	-	-	-	-	-	-	-	-	-	-	-	-	-	-	N.D.
		R	-	-	-	-	-	-	-	-	-	-	-	-	-	-	-	-	-	-	N.D.
	2	L	-	-	-	-	-	-	-	-	-	-	-	-	-	-	-	-	-	-	N.D.
		R	-	-	-	-	-	-	-	-	-	-	-	-	-	-	-	-	-	-	N.D.
	3	L	-	-	-	-	-	-	-	-	-	-	-	-	-	-	-	-	-	-	N.D.
		R	-	-	-	-	-	-	-	-	-	-	-	-	-	-	-	-	-	-	N.D.
	4	L	-	-	-	-	-	-	-	-	-	-	-	-	-	-	-	-	-	-	N.D.
		R	-	-	-	-	-	-	-	-	-	-	-	-	-	-	-	-	-	-	N.D.
	5	L	0	0	0	0	0	0	0	0	0	0	0	yes	0	0	0	0	0	0	**0**
		R	0	0	0	0	0	0	0	0	0	0	0	yes	0	0	0	0	0	0	**0**
	6	L	0	0	0	0	0	0	0	0	0	0	0	yes	0	0	0	0	0	0	**0**
		R	0	0	0	0	0	0	0	0	0	0	0	yes	0	0	0	0	0	0	**0**
	7	L	0	0	0	0	0	0	0	0	0	0	0	yes	0	0	0	0	0	0	**0**
		R	0	0	0	0	0	0	0	0	0	0	0	yes	0	0	0	0	0	0	**0**
Vehicle	8	L	-	-	-	-	-	-	-	-	-	-	-	-	-	-	-	-	-	-	N.D.
		R	-	-	-	-	-	-	-	-	-	-	-	-	-	-	-	-	-	-	N.D.
	9	L	-	-	-	-	-	-	-	-	-	-	-	-	-	-	-	-	-	-	N.D.
		R	-	-	-	-	-	-	-	-	-	-	-	-	-	-	-	-	-	-	N.D.
	10	L	-	-	-	-	-	-	-	-	-	-	-	-	-	-	-	-	-	-	N.D.
		R	-	-	-	-	-	-	-	-	-	-	-	-	-	-	-	-	-	-	N.D.
	11	L	0	0	0	0	0	0	0	0	0	0	0	yes	0	0	0	0	0	0	**0**
		R	0	0	0	0	0	0	0	0	0	0	0	yes	0	0	0	0	0	0	**0**
	12	L	0	0	0	0	0	0	0	0	0	0	0	yes	0	0	0	0	0	0	**0**
		R	0	0	0	0	0	0	0	0	0	0	0	yes	0	0	0	0	0	0	**0**

Supplementary Table 3. Ocular evaluation of uveitis scores at week 2 post-injection.

Week 2																					
			Anterior Segment											Posterior Segment							
			Conjunctiva			Cornea			Iris					Ocular Fundus							
Treatment	#Animal	Eye	Redness Congestion	Chemosis	Discharge	Opacity	Areas Involved	Cornea Pannus	Values	Aqueous Flare	Pupillary Light Reflex	Cellular Flare	Lens	Normal?	Vitreous Haze	Vitreous Cell	Optic Disc/Nerve	Retinal Vasculature	Retinal Pathology	Choroidal Pathology	Total Score
			(0-3)	(0-4)	(0-3)	(0-4)	(0-4)	(0-2)	(0-4)	(0-3)	(0-2)	(0-4)	(0-1)	Yes/No	(0-4)	(0-4)	(0-1)	(0-1)	(0-4)	(0-4)	
AAV7m8-CAG-GFP	1	L	1	0	0	0	0	0	0	0	0	0	0	yes	0	0	0	0	0	0	**1**
		R	1	0	0	0	0	0	0	0	0	0	0	yes	0	0	0	0	0	0	**1**
	2	L	1	0	0	0	0	0	1	1	1	1	0	yes	0	0	0	1	0	0	**6**
		R	1	0	0	0	0	0	1	1	1	0	0	yes	0	0	0	1	0	0	**5**
	3	L	1	0	0	0	0	0	1	1	1	0	0	yes	0	0	0	1	1	0	**6**
		R	1	0	0	0	0	0	1	1	1	0	0	yes	0	0	0	1	1	0	**6**
	4	L	0	0	0	0	0	0	1	1	1	0	0	yes	0	0	0	1	1	0	**5**
		R	0	0	0	0	0	0	1	1	1	0	0	yes	0	0	0	1	1	0	**5**
	5	L	0	0	0	0	0	0	0	0	0	0	0	yes	0	0	0	0	0	0	**0**
		R	0	0	0	0	0	0	0	0	0	0	0	yes	0	0	0	0	0	0	**0**
	6	L	0	0	0	0	0	0	0	0	0	0	0	yes	0	0	0	0	0	0	**0**
		R	0	0	0	0	0	0	0	0	0	0	0	yes	0	0	0	0	0	0	**0**
	7	L	0	0	0	0	0	0	0	0	0	0	0	yes	0	0	0	0	0	0	**0**
		R	0	0	0	0	0	0	0	0	0	0	0	yes	0	0	0	0	0	0	**0**
Vehicle	8	L	0	0	0	0	0	0	0	0	0	0	0	yes	0	0	0	0	0	0	**0**
		R	0	0	0	0	0	0	0	0	0	0	0	yes	0	0	0	0	0	0	**0**
	9	L	0	0	0	0	0	0	0	0	0	0	0	yes	0	0	0	0	0	0	**0**
		R	0	0	0	0	0	0	0	0	0	0	0	yes	0	0	0	0	0	0	**0**
	10	L	0	0	0	0	0	0	0	0	0	0	0	yes	0	0	0	0	0	0	**0**
		R	0	0	0	0	0	0	0	0	0	0	0	yes	0	0	0	0	0	0	**0**
	11	L	0	0	0	0	0	0	0	0	0	0	0	yes	0	0	0	0	0	0	**0**
		R	0	0	0	0	0	0	0	0	0	0	0	yes	0	0	0	0	0	0	**0**
	12	L	0	0	0	0	0	0	0	0	0	0	0	yes	0	0	0	0	0	0	**0**
		R	0	0	0	0	0	0	0	0	0	0	0	yes	0	0	0	0	0	0	**0**

Supplementary Table 4. Ocular evaluation of uveitis scores at week 4 post-injection.

Week 4																					
			Anterior Segment											Posterior Segment							
			Conjunctiva			Cornea			Iris					Ocular Fundus							
Treatment	#Animal	Eye	Redness Congestion (0-3)	Chemosis (0-4)	Discharge (0-3)	Opacity (0-4)	Areas Involved (0-4)	Cornea Pannus (0-2)	Values (0-4)	Aqueous Flare (0-3)	Pupillary Light Reflex (0-2)	Cellular Flare (0-4)	Lens (0-1)	Normal? Yes/No	Vitreous Haze (0-4)	Vitreous Cell (0-4)	Optic Disc/Nerve (0-1)	Retinal Vasculature (0-1)	Retinal Pathology (0-4)	Choroidal Pathology (0-4)	**Total Score**
AAV7m8-CAG-GFP	1*	L	-	-	-	-	-	-	-	-	-	-	-	-	-	-	-	-	-	-	-
		R	-	-	-	-	-	-	-	-	-	-	-	-	-	-	-	-	-	-	-
	2[#]	L	2	1	0	3	4	0	1	0	1	0	0	Not visible							**12**
		R	2	2	0	1	4	0	1	2	1	2	0	Not visible							**12**
	3[#]	L	2	2	0	1	4	0	1	2	1	2	0	Not visible							**15**
		R	2	1	0	2	4	0	1	2	1	2	0	Not visible							**15**
	4[#]	L	2	1	0	2	4	0	1	2	1	2	0	Not visible							**15**
		R	2	1	0	2	4	0	1	2	1	2	0	Not visible							**15**
	5[ǂ]	L	0	0	0	0	0	0	0	0	0	0	0	Yes	0	0	0	0	0	0	**0**
		R	0	0	0	0	0	0	0	0	0	0	0	Yes	0	0	0	0	0	0	**0**
	6[ǂ]	L	1	0	0	1	4	0	0	1	0	0	0	Yes	2	0	0	0	4	0	**13**
		R	1	0	0	1	4	0	0	1	0	0	0	Yes	2	0	0	0	4	0	**13**
	7[ǂ]	L	1	1	0	1	4	0	0	2	1	0	0	Not visible							**10**
		R	1	1	0	1	4	0	0	2	1	0	0	Not visible							**10**
Vehicle	8*	L	-	-	-	-	-	-	-	-	-	-	-	-	-	-	-	-	-	-	-
		R	-	-	-	-	-	-	-	-	-	-	-	-	-	-	-	-	-	-	-
	9[#]	L	0	0	0	0	0	0	0	0	0	0	0	yes	0	0	0	0	0	0	**0**
		R	0	0	0	0	0	0	0	0	0	0	0	yes	0	0	0	0	0	0	**0**
	10[#]	L	0	0	0	0	0	0	0	0	0	0	0	yes	0	0	0	0	0	0	**0**
		R	0	0	0	0	0	0	0	0	0	0	0	yes	0	0	0	0	0	0	**0**
	11[ǂ]	L	0	0	0	0	0	0	0	0	0	0	0	yes	0	0	0	0	0	0	**0**
		R	0	0	0	0	0	0	0	0	0	0	0	yes	0	0	0	0	0	0	**0**
	12[ǂ]	L	0	0	0	0	0	0	0	0	0	0	0	yes	0	0	0	0	0	0	**0**
		R	0	0	0	0	0	0	0	0	0	0	0	yes	0	0	0	0	0	0	**0**

** Animals sacrificed on day 15; # animals sacrificed on day 24;* ǂ animals sacrificed on day 28.

Supplementary Table 5. Detail of the number of porcine aqueous and vitreous humor samples used for pro-inflammatory factor analyses.

Nº Eyes per sample	Vehicle	AAV Baseline	AAV Week 1	AAV Week 2	AAV Week 4 (cohort 1)	AAV Week 4 (Cohort 2)
**Aqueous**	8	8	8	8	6	4
**Vitreous**	6	N.D.	N.D.	2	3	4

Supplementary Table 6. Antibodies used for IF, MSD and flow cytometry.

**Antibodies for MACSima imaging**	**Dilution**	**Source**	**Catalog number**
Phospho-Histone H2A.X (Ser139) Monoclonal Antibody (clone CR55T33), PE	1:50	Thermo Fisher	12-9865-42
Alexa Fluor® 647 Anti-Iba1 antibody (clone EPR16588)	1:50	Abcam	ab225261
SLA Class II DR Monoclonal Antibody (clone 2E9/13), FITC	1:50	Thermo Fisher	MA5-16490
GFAP Antibody, anti-human/mouse/rat, PE, REAfinity™	1:50	Miltenyi Biotec	130-118-351
Brn-3a/BRN3A/POU4F1 Antibody (14A6)	1:50	Santa Cruz	sc-8429
Cleaved Caspase-3 (Asp175) Antibody	1:200	Cell Signaling	9661
Factor IX Polyclonal Antibody – Affinity Purified – FITC Conjugated	1:50	Affinity biologicals	GAFIX-APFTC
**Antibody kits for MSD capture and detection**	Concentration	Source	Catalog number
Swine IL-6 ELISA	1 µg/ml	King Fisher Biotech	DIY0727S
Swine CXCL10 (IP-10) ELISA	2 µg/ml	King Fisher Biotech	DIY0723S
Swine CCL3L1 ELISA	1 µg/ml	King Fisher Biotech	DIY0720S
Swine CCL2 (MCP-1) ELISA	1 µg/ml	King Fisher Biotech	DIY0719S
**Antibodies for flow cytometry**	Dilution	**Source**	**Catalog number**
**Mouse studies**			
Brilliant Violet 421™ anti-mouse CD45 Antibody, clone 30-F11	1:200	Biolegend	103133
Alexa Fluor® 700 anti-mouse CD3 Antibody, clone 17A2	1:200	Biolegend	100216
Brilliant Violet 785™ anti-mouse CD4 Antibody, clone RM4-5	1:200	Biolegend	100551
APC/Cyanine7 anti-mouse CD8a Antibody, clone 53-6.7	1:200	Biolegend	100714
PE/Dazzle™ 594 anti-mouse CD19 Antibody, clone 6D5	1:200	Biolegend	115554
Brilliant Violet 785™ anti-mouse Ly-6C Antibody, clone HK1.4	1:200	Biolegend	128041
PE/Dazzle™ 594 anti-mouse CD11c Antibody, clone N418	1:200	Biolegend	117348
PerCP/Cyanine5.5 anti-mouse/human CD11b Antibody, clone M1/70	1:200	Biolegend	101228
TruStain FcX™ PLUS (anti-mouse CD16/32) Antibody	1:200	Biolegend	156604
**Pig studies**			
CD45 Monoclonal Antibody (clone K252.1E4)	1:200	Thermo Fisher	MA5-28391
SLA Class II DR Monoclonal Antibody (clone 2E9/13)	1:200	Thermo Fisher	MA5-28503
PerCP/Cyanine5.5 anti-mouse/human CD11b Antibody (clone M1/70)	1:200	Biolegend	101228
PE anti-human CD135 (Flt-3/Flk-2) (clone BV10A4H2)	1:200	Biolegend	313305
BD Pharmingen™ PerCP-Cy™5.5 Mouse Anti-Pig CD3ε	1:200	BD Biosciences	561478
Fc Receptor Binding Inhibitor Polyclonal Antibody, eBioscience™	1:5	Thermo Fisher	14-9161-73
